# Nanoparticles coated by chloramphenicol in hydrogels as a useful tool to increase the antibiotic release and antibacterial activity in dermal drug delivery

**DOI:** 10.1007/s43440-023-00482-4

**Published:** 2023-04-11

**Authors:** Dawid Bursy, Radosław Balwierz, Paweł Groch, Paweł Biernat, Adam Byrski, Katarzyna Kasperkiewicz, Wioletta Ochędzan-Siodłak

**Affiliations:** 1 Department of Drug Forms Technology, Faculty of Pharmacy, Wrocław Medical University, Borowska St. 211, 50-556 Wrocław, Poland; 2 Institute of Chemistry, University of Opole, Oleska St. 48, 45-052 Opole, Poland; 3 Institute of Metallurgy and Materials Science, Polish Academy of Sciences, Reymonta St. 25, 30-059 Cracow, Poland; 4 Faculty of Natural Sciences, Institute of Biology, Biotechnology and Environmental Protection, University of Silesia in Katowice, Jagiellońska St. 28, 40-032 Katowice, Poland

**Keywords:** Nanoparticles, Silica nanoparticles, Gold nanoparticles, Carbopol, Chloramphenicol, Drug delivery, Bacteriostatic activity, Antibiotics, Rheological properties

## Abstract

**Background:**

Nanocarriers for antibacterial drugs became hopeful tools against the increasing resistance of bacteria to antibiotics. This work focuses on a comprehensive study of the applicability and therapeutic suitability of dermal carbopol-based hydrogels containing chloramphenicol carried by various nanoparticles (AuNPs and SiNPs).

**Methods:**

The different forms of carbopol-based drugs for dermal use were obtained. Five different concentrations of chloramphenicol and two types of nanoparticles (silica and gold) in carbopol-based ointments were tested. The influence of different carbopol formulations with nanocarriers on the rheological properties as well as the release profile of active substances and bacteriostatic activity on five reference strains were determined.

**Results:**

The properties of the obtained hydrogels were compared to a commercial formulation, and finally it was possible to obtain a formulation that allowed improved antimicrobial activity over a commercially available detreomycin ointment while reducing the concentration of the antibiotic.

**Conclusion:**

The work indicates that it is possible to reduce the concentration of chloramphenicol by four times while maintaining its bacteriostatic activity, which can improve the patient’s safety profile while increasing the effectiveness of the therapy.

**Graphical abstract:**

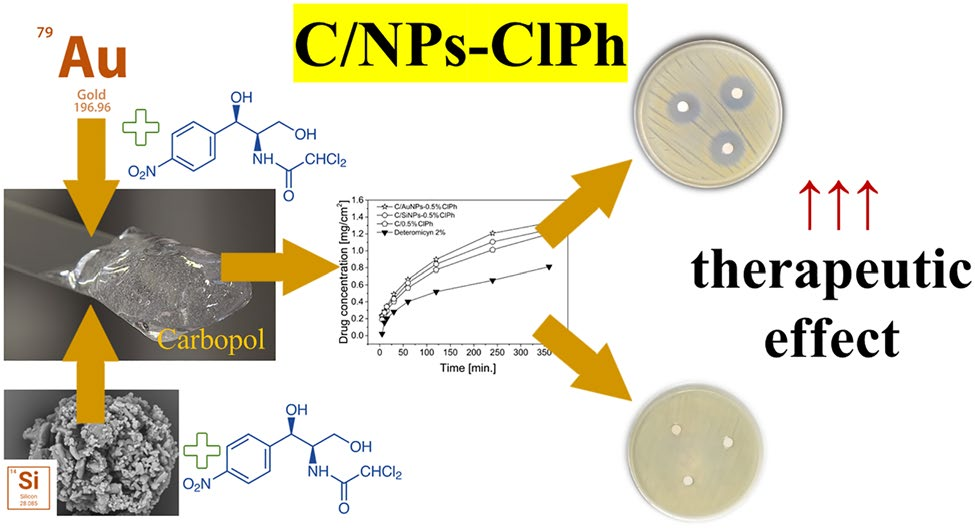

**Supplementary Information:**

The online version contains supplementary material available at 10.1007/s43440-023-00482-4.

## Introduction

Nanoparticles (NPs), due to their size, are characterised by different properties compared to the macroscopic material (differences include specific surface area, surface energy, and pore size) [1]. NPs are divided into organic (dendrimers, liposomes and micelles), inorganic: metal nanoparticles (e.g. gold, silver, copper, aluminium, cadmium, lead nanoparticles) and from metal and non-metal oxides (e.g. iron(III) oxide, aluminium oxide, silicon(IV) oxide, titanium(IV) oxide). There is also a separate category, carbon nanoparticles, consisting only of carbon atoms (fullerenes, graphene, nanofibers and nanotubes) [2]. The considerable interest in these materials can be evidenced by the annual number of published papers in JCR-indexed journals related to nanoparticles (about 2.500 in 2022) in the PubMed database alone.

Nanoparticles have recently attracted considerable interest in academia and industries, including gold nanoparticles (AuNPs) and silica nanoparticles (SiNPs), which have more favourable physicochemical properties and higher biological activity than metallic gold and silica [3].

It should be noted that AuNPs are characterised by small size (in the range of a few to a few hundred nanometres), high specific surface area relative to volume, ease of synthesis with the ability to control the parameters of the final product with the desired sizes and shapes which encourages the search for new applications in diagnostics and therapeutics [4]. In addition, the AuNPs are biocompatible and exhibit low toxicity, which is especially important when administered *per os* [5] Several methods of AuNPs synthesis have been described in the literature, including the reduction of Au^3+^ or Au^+^ ions [6], Turkevich’s method [6, 7], utilizing citric acid or sodium bromide as a reducing agent [8, 9] as well as the Brust–Schiffrin method [10]. The reduction of chloroaurate acid with ascorbic acid is also described [11], as well as the so-called Seeding-Growth method, which leads to the formation of gold nanotubes [8, 12]. Microorganisms are also being used to produce gold nanoparticles [5, 13], fungi [14], plants [15, 16] and algae [5, 17–19].

The obtained AuNPs are used as drug carriers, e.g. for methotrexate and doxorubicin. AuNPs as a carrier for active pharmaceutical ingredients (API) show increased effectiveness in anticancer therapy by accumulating nanoparticles with the active drug in cancer tissues [20]. The attachment of bleomycin and doxorubicin to carrier AuNPs [21] showed high stability of the conjugates and specificity of action against tumour cells, achieving identical cytotoxic activity, compared to the administration of bleomycin and doxorubicin without a carrier at lower doses. Similar results were obtained by combining gold nanoparticles with folic acid and chlorambucil [22].

It should be noted that AuNPs, as a carrier for antibacterial drugs, are a hopeful tool against the increasing resistance of bacteria to antibiotics. The combinations of antibiotics with nanoparticles are used to increase the drug’s potency and/or to reduce its toxicity. It is considered that the gold nanoparticles coated with the antibiotic can more easily penetrate through the membrane of bacterial cells and, at the same time, exhibit a protective effect against the antibiotic molecule (e.g., protecting against bacterial enzymes such as *β*-lactamase) [23] It was shown that for vancomycin-resistant *Enterococcus* (VRE) and vancomycin-resistant *Streptococcus aureus* (VRSA) strains, attachment to nanoparticles of vancomycin reduced the MIC from 16 to 32 times and eight times, respectively, compared to the use of unbonded vancomycin [24] The beneficial effects of gold nanoparticles were also demonstrated when the drug was applied to the skin. The use of nanoparticle conjugates with gentamicin in streptococcal infection in animals showed that accumulation of the drug at the site of infection was noted more than with the free antibiotic. Similarly, the application of fluorouracil nanoparticle conjugates exhibited twice the absorption of the therapeutic substance from the cream with fluorouracil nanoparticle conjugates relative to the cream without the addition of nanoparticles [25] The cream and gel with gold nanoparticles combined with fluorouracil resulted in an approximately 18-fold and sevenfold tumour volume reduction compared to the control trial [26] In addition, gold nanoparticles are used in the photothermic treatment of cancer [27, 28] and diagnostic imaging [29–31].

The silicon nanoparticles (SiNPs) belong to the second group of nanoparticles that are relatively simple to synthesise and modify their physicochemical properties. The SiNPs are characterised by a porous structure that can be used as a potential storage and/or carrier for active substances [32]. The primary method of SiNPs synthesis is a chemical method based on the hydrolysis reaction of a silica precursor with the formation of spherical structures. Of all the preparation methods, the ones attracting the most attention are the Stöber synthesis, the precipitation method, and the microemulsion method [33]. In pharmaceutical practice, physical adsorption and solvent evaporation are the two main strategies for forming connections between SiNPs and drugs [34, 35]. SiNPs were used to attach polymyxin B and vancomycin [36], as well as rifampicin [37] resulting in improved antimicrobial activity.

What seems to be particularly interesting is the inclusion of nanoparticles in non-lipophilic dermatological drugs used in the form of hydrogels. Hydrogels can provide numerous therapeutic benefits, including controlled release of the active ingredient (for both large-molecule and small-molecule drugs) and protection of unstable (environmentally sensitive) drugs [38]. Many advanced hydrogels have been developed, each with different and/or unique characteristics, such as high swelling capacity, enhanced oxygen permeability, biocompatibility, ease of API loading and release, and structural diversity forms [39, 40]. Hydrogels are a drug form used to administer painkillers [40] or antibiotics and may exhibit antibiotic properties [41].

This drug form can be used to apply chloramphenicol (ClPh), which seems to be a forgotten antibiotic, on which modern research concertedly focuses only to obtain a form for application to the eye (i.e., eye ointment) [42], and the emerging antibiotic resistance in the world is causing renewed interest in its use. The ClPh is an antibiotic obtained synthetically and is included in the group of bacteriostatic antibiotics [43, 44]. Indications for internal use are the treatment of severe infections such as cholera, meningitis, typhoid fever and rickettsiosis [42, 45, 46]. ClPh in the pharmacy in Poland is available only in the form of a dermatological preparation (1% and 2% ointment based on vaseline). Used in ointment form applied 2–3 times a day (every 6–8 h) for no longer than 14 days [45–47]. The use of 2% ClPh ointment for more than 14 days or on an extensive area of the skin increases the risk of severe side effects of chloramphenicol, like grey baby syndrome [46–48]

This work focuses on a comprehensive study of the assessment of the applicability and therapeutic suitability of dermal carbopol-based hydrogels containing chloramphenicol carried by various nanoparticles (AuNPs and SiNPs). It should be noted that the literature lacks both scientific papers on the combined use of chloramphenicol carried by nanoparticles on hydrogel substrates and the determination of rheological properties (storage modulus (Gʹ), frequency sweep was carried out using an oscillatory-rotational rheometer). In addition, the influence of different formulations on the release profile and bacteriostatic activity was also determined. It should be highlighted that bacteriostatic activity was performed using the *Escherichia coli* ATTC 8739, *Staphylococcus aureus* ATTC 6538P, *Pseudomonas aeruginosa* ATTC 27853 and *Bacillus subtilis* ATTC 6633 reference strains, as well as the *Candida albicans* ATTC 10231 fungal strain. In addition, the properties of the obtained hydrogels were compared to a commercial formulation, finally obtaining a formulation that allowed improved antimicrobial activity over a commercially available detreomycin formulation while reducing the concentration of the antibiotic.

## Materials and methods

### Reagents

Tetraethoxysilane-TEOS 99.99% (Aldrich Chemistry, Darmstadt, Germany) (78-10-4); water ammonia solution 25% (Chempur, Piekary Slaskie, Poland) (1336-21-6); anhydrous ethanol (46.07 g/mol, JT Baker, Phillipsburg, NJ, USA) (64-17-5); purified water (obtained with Polwater deionizer CNX-100 T 717); sodium hydroxide (Chempur, Piekary Slaskie, Poland) (1310-73-2); gold chloride(III) (303,33 g/mol, Aldrich Chemistry, Darmstadt, Germany) (13453-07-1), ascorbinic acid (176,12 g/mol, Aldrich Chemistry, Darmstadt, Germany) (50-81-7), chloramphenicol FPV (PPH Galfarm, Cracow, Poland) (56-75-7); and carbopol 934P (BF Goodrich, Cleveland, OH, USA) (9003-01-4).

### Silica nanoparticles synthesis

Silica carrier and chloramphenicol-coated silica carriers were prepared according to the methodology described by Balwierz et al. [49], using material A, described in the publication as the base for coating with chloramphenicol. The ratio of chloramphenicol used to coat the silica nanoparticles was modified in the current paper. It was two parts by weight of chloramphenicol to one part by weight of silica nanoparticles.

### Nanogold particles synthesis

The synthesis was carried out using a method effective for ascorbic activity with gold(III) chloride [11]. It used 8 parts of 0.0005 mol/L gold(III) chloride solution and 4 parts of 0.01 mol/L ascorbic acid solution for 24 parts of water.

In a 500 mL glass bottle, 400 mL of the final compound was prepared; for this purpose, an appropriate volume of gold(III) chloride solution and deionised water were mixed, and then the bottle was placed in an ultrasonic bath. The ultrasound was turned on simultaneously, along with a single addition of ascorbic acid. The synthesis was carried out for one hour using a continuous ultrasound program. The initial running temperature was 33 °C and increased gradually throughout the synthesis, reaching a final value of 67 °C in the last minute of the running synthesis.

In the next step, ethanolic solutions of chloramphenicol were prepared at concentrations of 0.125 g/100 mL (the ratio of Au attachment to ClPh was 1:38 w/w), 0.250 g/100 mL (1:76 w/w) and 0.500 g/100 mL (1:152 w/w). 100 mL of each solution containing gold nanoparticles was measured into three round-bottom flasks, then the appropriate chloramphenicol solution was added, and the solvent was removed. Evaporation of the solvent was carried out at 65 °C under 200 millibar pressure until about 70 mL of solution remained in the flask. The solutions were allowed to cool at room temperature and then made up to the mark in a 100 mL volumetric flask with deionised water to obtain 100 g of the compound. The resulting gold nanoparticles coated with chloramphenicol were used for further studies.

### Size measurement of gold and silica nanoparticles after coating with chloramphenicol

The SiNPs, AuNPs, SiNPs-ClPh and AuNPs-Clph particle size was estimated by Dynamic Light Scattering (DLS) using the Malvern Zetasizer Nano-ZS ZEN 3600 device (Software: v7.13, Worcestershire, UK) in accordance with the reference [50]. The samples of SiNPs, AuNPs and SiNPs-ClPh and AuNPs-ClPh were prepared by weighing 1 mg of powder crushed in an agate mortar and suspending it in 2 mL of deionised water by shaking in an ultrasonic bath for 5 min to disintegrate the aggregates.

### Hydrogel preparation

To compare the efficiency of the obtained nanoparticles against the commercially available form, hydrogels with chloramphenicol-coated nanoparticles (AuNPs and SiNPs) were prepared. The obtained nanoparticles coated with antibiotic and crystalline chloramphenicol (C/2.0%ClPh) were resuspended in carbopol gel to obtain the assumed concentrations (0.125% w/w, 0.25% w/w, 0.50% w/w and/or 2.0% w/w). Gels were prepared using 1.0% Carbopol 934P according to the references [51, 52]. Carbopol was mixed with silica carrier (SiNPs) or nanogold (AuNPs) coated with chloramphenicol and crosslinked with a 10% sodium hydroxide solution. The gels prepared this way were transferred to a refrigerator for 24 h for degassing. The finished gels were stored at 8 °C until further use.

### Content of the chloramphenicol in the carbopol gel

The chloramphenicol content in the carbopol gel was determined by high-performance liquid chromatography (HPLC) according to the methogolody presented by Balwierz [49]. The methodology is extensively described in the supplementary materials.

### The release study of chloramphenicol

The release study of chloramphenicol from the nanocarriers was performed in a Hanson Research SR8PLUS pharmacopeial paddle dissolution apparatus with Enhancer cell vessels with an Agilent 850DS autosampler according to references [49]. The acceptor medium in the study was borate buffer isotonic with sodium chloride, pH equal to 7.2. The composition of the buffer: 11.0 g boric acid, 2.0 g sodium tetraborate, and 2.0 g sodium chloride supplemented to 1000.0 g with purified water. The study was conducted using two formulations obtained from respective syntheses of chloramphenicol-coated nanosilica particles (SiNPs-0.50%ClPh) and nanogold particles (AuNPs-0.50%ClPh) against two reference formulations prepared from chloramphenicol without a carrier (C/2.0%ClPh) and a commercial 2% chloramphenicol ointment produced by Chema-Elektroment (permit No. R/3286) (Detreomycin-2%), six units for each formulation. The methodology is extensively described in the supplementary materials.

### Antimicrobial susceptibility testing

For microbiology studies, four bacteria strains were selected: *Escherichia Coli* ATTC 8739, *Staphylococcus aureus* ATTC 6538*P*, *Pseudomonas aeruginosa* ATTC 27853 and *Bacillus subtilis* ATTC 6633. Additionally, the *Candida albicans* ATTC 10231 fungal strain was used. These strains are well described in the literature and often used in susceptibility testing. Prior to tests, microorganisms were cultivated overnight at (36 ± 1)°C using media presented in Table 1, as recommended by the manufacturer:

**Table 1:** Culture media used to culture microorganisms

Item	Microorganism	Culture media
1	*Escherichia coli* ATTC 8739	Nutrient broth
2	*Staphylococcus aureus* ATTC 6538*P*	Trypticase soy broth
3	*Pseudomonas aeruginosa* ATTC 27853	Trypticase soy broth
4	*Bacillus subtilis* ATTC 6633	Brain heart infusion broth
5	*Candida albicans* ATTC 10231	YPD broth

### Disk diffusion test

The inoculum turbidity was adjusted to 0.5 McFarland standard (approx. OD_600nm_ = 0.06) by diluting overnight cultures in phosphate-buffered saline (PBS). The test was performed using the Mueller Hinton Agar. Plates were struck to form a bacterial lawn. The diameter of the disks used in the experiment was 6 mm. Each disc was loaded with (12 ± 0.1) mg of the tested gel. Plates were inverted and incubated at (36 ± 1)°C for 24 h in a humid (~ 90%) atmosphere. Thereupon, the diameter of clear zones was measured.

### Bacterial growth kinetics

The inoculum turbidity was adjusted to 0.5 McFarland standard by diluting overnight cultures in Mueller Hinton Broth. Inoculum turbidity was corrected by OD_600nm_ value of MH Broth. The test was performed by mixing 150 μL of inoculum and 450 μL of gel in 24-well polystyrene cell culture plates (NEST^®^Scientific). A mixture of 150 μL of inoculum and 450 μL PBS was used as a reference. Plates were incubated at (36 ± 1)°C for 24 h with an orbital shaking of 5-mm amplitude and frequency of 120 rpm in TECAN Spark^®^ 20 M. Every 60 min, the absorbance signal was read at 600 nm.

### Rheological investigation

The rheological investigation was performed using a strain-controlled Discovery Hybrid Rheometer HR20 (Ta Instruments) equipped with a 40 mm parallel plate with a gap of 1 mm. A special measuring cell was employed to prevent water evaporation and ensure temperature stabilisation. Motor and geometry calibrations were performed prior to each experiment. The samples were prepared at least 2 h before each experiment to allow for complete gelation. Each sample in sol state was transferred onto the rheometer plate (heated to 32 °C) and subsequently lowered the geometry to the desired gap, and the measuring chamber was closed. After loading, the samples were equilibrated to the set temperature for at least 8 min. All experiments were performed at 32 °C according to the procedure for dermal systems. To determine the value of the storage modulus (*Gʹ*) of the materials obtained during the gelation process, the measurements were performed in oscillation mode using a frequency of 1 Hz and a strain in the range of 10^–3^–200%. In turn, the frequency sweep experiments were performed at *γ* = 1% (within the linear viscoelastic regime as determined from strain sweep experiments) for *ω* = 628–0.01 rad/s.

### The analysis of release profiles and statistical analysis

Based on the pharmaceutical availability tests conducted for each of the six units of the drug formulation belonging to the four formulations being compared, the release profile was determined in terms of the percentage of active substance released and the absolute value of the rate of permeation per unit area. The process constant was calculated for the total rate concerning the Higuchi kinetic model. For the four parameter values determined, the basic descriptive statistics were first calculated: deviation (SD) and standard error (SE), coefficient of variation (CV, RSD) and 95% significance interval for the mean (95%CI). Next, the normality of their distributions was assessed using the W Shapiro–Wilk test. Finally, Levene’s test was used to assess the homogeneity of variance. All analysed variables met the assumptions of both normalities of distribution and homogeneity of variance. At no time point did the determined coefficients of variation exceed the CV = 10% limit defined by FDA and EMA guidelines. In the next step, the statistical significance of differences between the mean values of the compared parameters was evaluated using parametric analysis of variance ANOVA. For post-hoc multiple comparisons, Fisher’s LSD (least significant difference) test was applied. A significance level of *α* = 0.05 was used in all analyses, and statistical tests were applied.

A parametric analysis of variance ANOVA was used to compare microbial inhibition zone values. For post-hoc multiple comparisons, Fisher’s LSD test was used. In all analyses and statistical tests performed, a significance level of *α* = 0.05 was adopted.

## Results and discussion

### Rheological behaviour of hydrogels

**Fig. 1:**
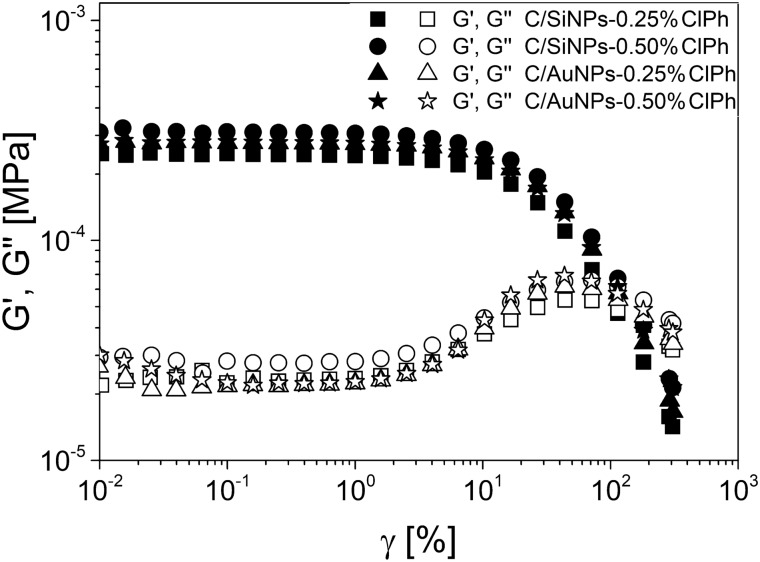
Storage and loss modulus (*G′, G′′*) as a function of strain (*γ*) at 32 °C for obtained hydrogels. The rheological properties were measured by a shear rheometer

**Fig. 2:**
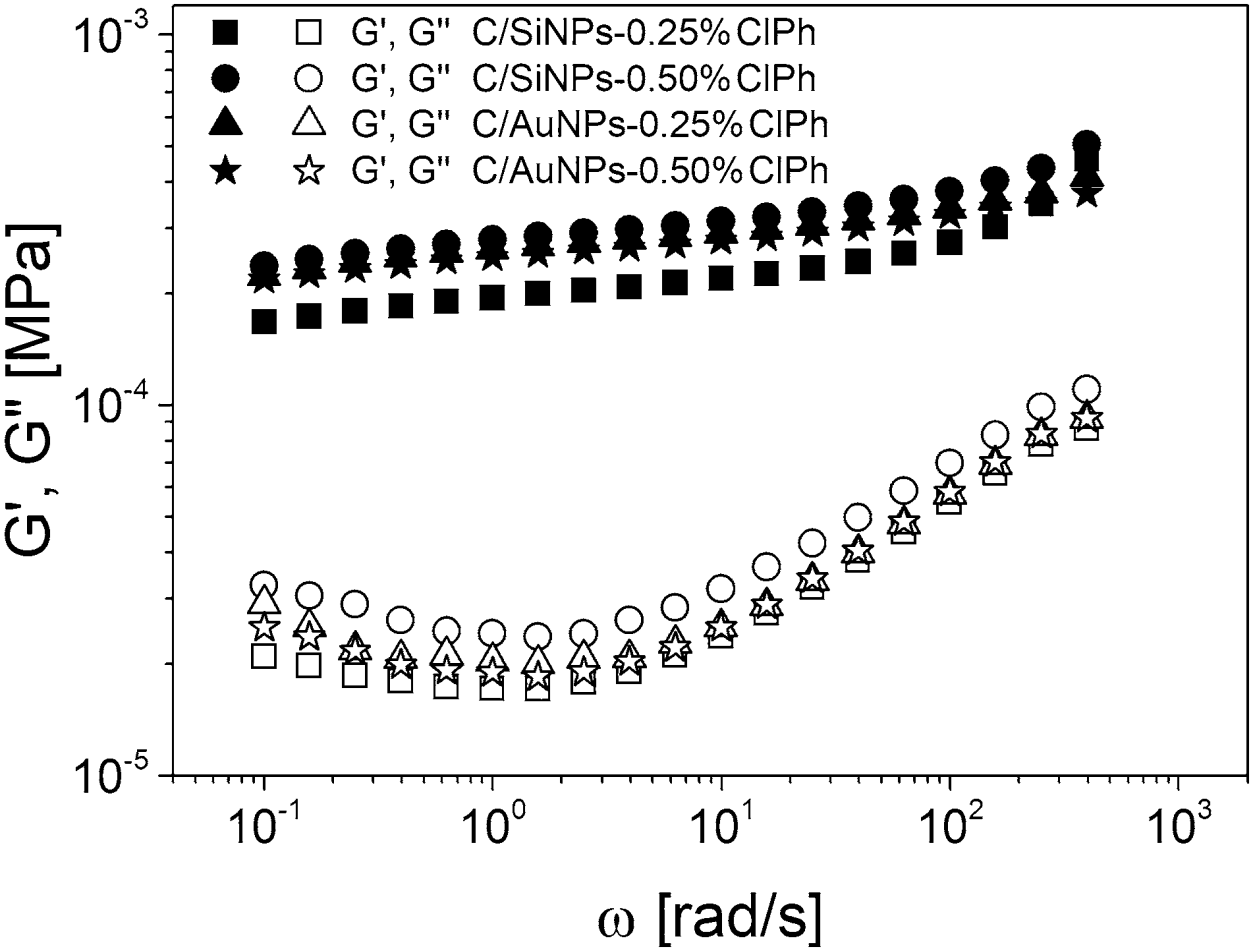
Selected storage and loss modulus (*G′, G′′*) as a function of oscillation frequency (*ω*) within the linear viscoelastic regime (*LVE*) at 32 °C for obtained hydrogels. The rheological properties were measured by a shear rheometer

**Fig. 3:**
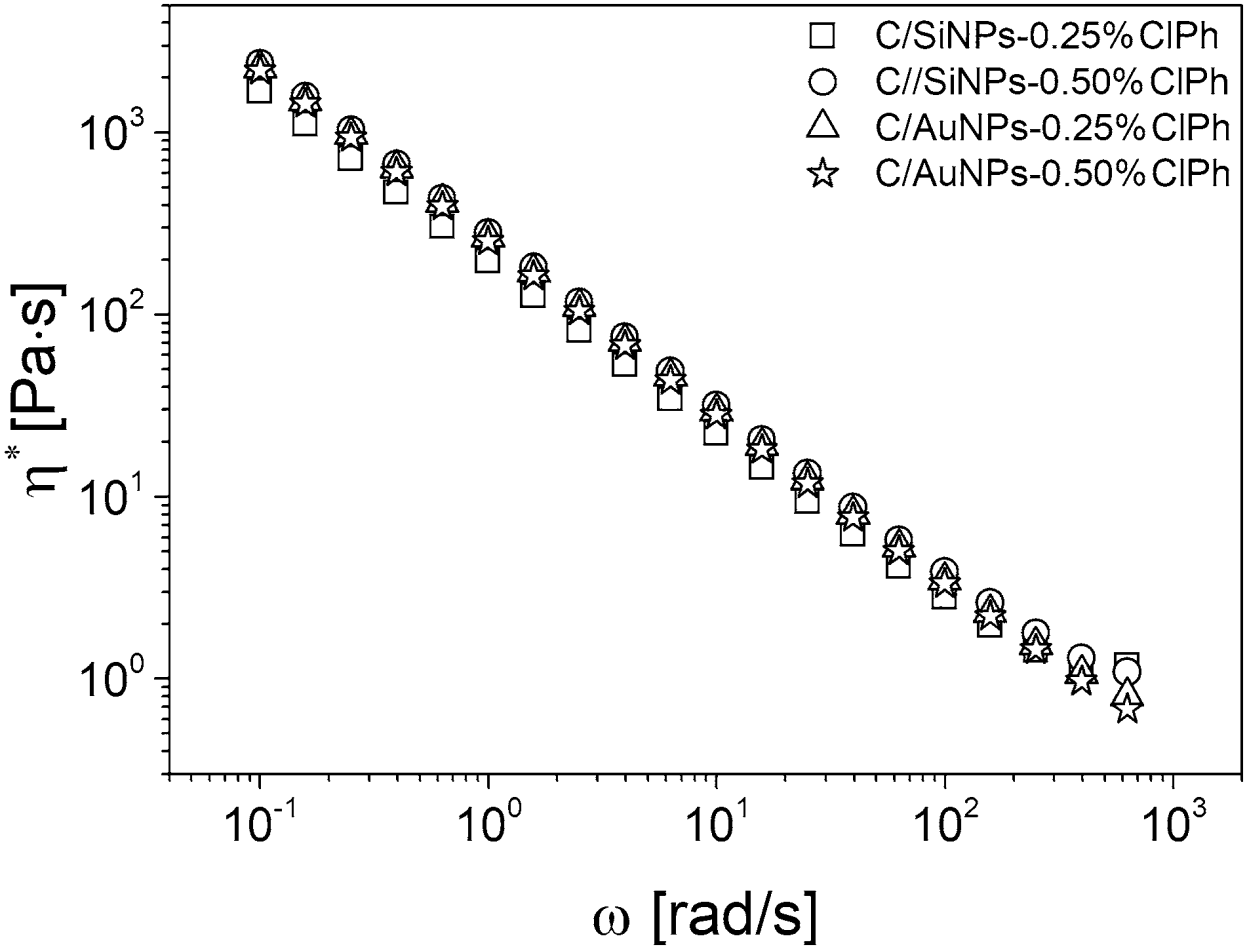
Complex viscosity (η*) as a function of the oscillation frequency (*ω*) within the linear viscoelastic regime (*LVE*) at 32 °C for obtained hydrogels. The rheological properties were measured by a shear rheometer

The strain and frequency-dependent rheology was employed to assess the viscoelastic properties of carbopol hydrogel filled with chloramphenicol immobilised on solid supports**/**silica gel and gold nanoparticles (Figs. 1, 2 and 3).

The investigated hydrogels exhibited linear viscoelastic (LVE) region up to 2% of oscillation strain (*γ)*, where storage modulus (*G′)* remained unchanged. The *G'* value gradually decreased above *γ* = 2% while above *γ* = 5%, followed by a sharp decrease of elastic shear modulus denoting the non-linear viscoelastic region (Fig. 1) [53].

It should be noted that the linear viscoelastic region (LVE) of hydrogels was dependent on the kind of carrier used. It was found that the carbopol hydrogels with chloramphenicol/gold (AuNPs/ClPh) nanoparticles were characterised by a higher shear strain (*γ*_*L*_) in comparison to carbopol hydrogel with chloramphenicol/silica gel (SiNPs/ClPh), what is associated with a strong interaction of a polar group of chloramphenicol/gold nanoparticles with hydrogel (Table 2). This phenomenon can be explained by a higher AuNPs/ClPh molar ratio compared to SiNPs/ClPh (Section “Silica nanoparticles synthesis”). Additionally, the *γ*_*L*_ value slightly increased with increasing content of drug immobilised on silica gel incorporated into the hydrogel network in contrast to the Carbopol hydrogel with chloramphenicol/gold nanoparticle (Table 2).

**Table 2:** The rheological parameters of obtained hydrogel

Item	Sample	*γ*_*L*_ [%]	*G*_*N*_^*0*^ [Pa]	*ξ* [nm]	*ρ* [µmol/cm^3^]
1	C/SiNPs-0.25%ClPh	2.91	199.7	27.6	0.079
2	C/SiNPs-0.5%ClPh	2.96	292.3	24.3	0.115
3	C/AuNPs-0.25%ClPh	4.16	279.7	24.7	0.110
4	C/AuNPs-0.5%ClPh	3.14	256.5	25.4	0.101

*C/* carbopol based formulation, *AuNPs* gold nanoparticles, *SiNPs* silica nanoparticles, *ClPh* chloramphenicol, *γ*_*L*_ shear strain, *G*_*N*_^*0*^ plateau modulus, *ξ* network mesh sizes, ρ mesh density

It is worth noting that after exceeding the constant value in the LVE region for investigated samples, the loss modulus (*G"*) increased sharply until (stage I), after reaching a maximum (stage II), the curve again decreased steeply. These results indicated that a few individual bonds in the polymer network rupture due to shear strain, which led to the formation of microcracks (stage I). However, the entire surrounding material still remained firmly together [54]. The *G"* value increased with increasing *γ* value due to the conversion of the deformation energy into friction heat by freely movable fragments around the micro-cracks, which are not integrated within the hydrogel network. Moreover, as the individual microcracks grow further, they eventually form continuous macrocracks that run through the entire sample (stage II).

It was found that the incorporation of chloramphenicol/silica (SiNPs/ClPh) gel into the hydrogel network resulted in a shift in stage II toward higher shear strain in contrast to the counterpart with chloramphenicol/gold nanoparticles (AuNPs/ClPh). These results suggested that the chloramphenicol/silica gel in hydrogels was characterised by lower susceptibility to macrocrack formation, which is associated with an uninterrupted hydrogel coating at the site of the wound.

An isothermal frequency sweep analysis was also carried out to understand the relationship between filler species incorporated into the hydrogel network and the rheological response (Fig. 2).

The hydrogel containing chloramphenicol/silica gel revealed the typical behaviour of the crosslinked network, where the hydrogel behaved predominantly as solid due to the storage modulus being much higher than the loss modulus in all ranges of oscillation frequency. Hydrogel networks were highly elastic, and the almost constant *G'* value indicated that dissipation of energy deformation was negligible in each cycle of oscillation; therefore, it is concluded that there are few free ends or loops that are considered defects in the crosslinked network [54, 55] In addition, the crossover point was not observed, which is associated with the fact that carbopol-based hydrogel was a highly crosslinked polymer and most likely consisted largely of simple long-chain polymer molecules with excessively entangled chains.

Furthermore, the effective network mesh sizes (*ξ*) were estimated using the equation: *G*_*N*_^*0*^ = *kT*/*ξ* [55] and are presented in Table 2. The *ξ* values for C/SiNPs-ClPh hydrogels fell within the 24.3–27.6 nm range and were generally higher in contrast to the network mesh size values for C/AuNPs-ClPh hydrogels, which ranged from 24.7 up to 25.4 nm. It was found that the *ξ* values for C/SiNPs-ClPh hydrogels decreased with the filler content increasing from 27.6 nm to 24.3 nm, suggesting the presence of molecular rearrangements due to the effect of hydrogen bonds between silica gel and the hydrogel network. The opposite behaviour was observed in the case of C/AuNPs-ClPh hydrogels, where *ξ* values increased with the increase of AuNPs/ClPh content. This result can be explained by the absence of additional intermolecular bonding between gold nanoparticles with the hydrogel network, which caused the *ξ* values increase. It can be concluded that the hydrogel networks with higher *ξ* values will allow for faster and easier release of the solvent with chloramphenicol entrapped in the molecular folds to the skin’s surface. It should be noted that the tunable design of such systems, with different pore sizes, can be achieved by the kind and content of filler incorporated into the hydrogel network.

Further insight into the molecular networks was obtained from the dependence of the complex viscosity (η^*^) with the frequency of oscillation (Fig. 3).

The results showed that the viscosity of the hydrogel networks decreased with the increasing shear rate, referred to as shear thinning. It should be highlighted that shear thinning is a desired viscoelastic behaviour in formulations designed for application to the skin’s surface. For example, when applying hydrogels at a high frequency of the shear, the material will flow easily, allowing for a successful distribution on the skin surface, and then it will restore its molecular structure and persist on the skin surface for a prolonged period. It was found that higher η^*^ values were observed for C/AuNPs-ClPh hydrogels compared to C/SiNPs-ClPh hydrogels (Fig. 3).

Moreover, the complex viscosity was consistent with the scaling η^*^∼ *ω*^*−n*^, where *n* = − 1 is attributed to an ideal chemically crosslinked network [56]. The fit to the experimental data gave the exponent − 0.92, − 0.93 for C/AuNPs-0.25%ClPh and C/AuNPs-0.50%ClPh, as well as − 0.88, − 0.91 for C/SiNPs-0.25%ClPh and C/SiNPs-0.50%ClPh, respectively. Therefore, it is concluded that the C/AuNPs-ClPh networks have minor defects (dangling ends and loops), whereas the C/SiNPs-ClPh networks contain a higher content of defects that are the source of mechanical energy dissipation in comparison to C/AuNPs-ClPh hydrogels.

### Study of the release of chloramphenicol from the carbopol-based gel

**Fig. 4:**
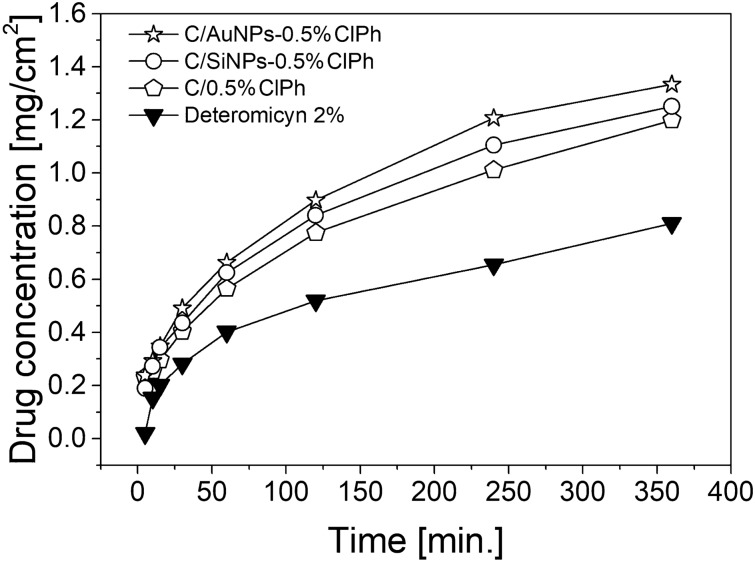
The amount of released chloramphenicol from the tested hydrogels and commercial preparation after conversion to the surface area [mg/cm^2^] in time [min]. The release study of chloramphenicol from the silica carrier was performed in a Hanson Research SR8PLUS pharmacopeial paddle dissolution apparatus with Enhancer cell vessels. The release profile was determined in terms of the percentage of active substance released and the absolute value of the rate of permeation per unit area. The statistical significance of differences between the mean values of the compared parameters was evaluated using parametric analysis of variance ANOVA. For post-hoc multiple comparisons, Fisher’s NIR LSD test was applied. Results are presented in supplementary materials (Tables S2, S3 and Figure S2). Abreviations: C/0.5%ClPh—carbopol formulation based only on chloramphenicol (without the silica or nanogold carriers); C/SiNPs-0.5%ClPh—carbopol formulation based on SiNPs carrier and chloramphenicol; C/AuNPs-0.5%ClPh—carbopol formulation based on AuNPs carrier and chloramphenicol

To evaluate the release profile of chloramphenicol from the prepared hydrogel formulations containing nanoparticles (AuNPs and SiNPs) coated with the antibiotic and the antibiotic without nanoparticles (C/0.5%ClPh) against commercially available detreomecin (Detromycin-2%), a study of the release of the active ingredient from the prepared formulations was performed. Higuchi’s model based on Fick’s law was used to describe the release profiles, which considers the percentage released as a linear relationship with the time element. This model is most often used when comparing different modified-release formulations. Hydrogel formulations are considered to be such [57] The chloramphenicol content of the tested formulations was as expected, and the size of the nanoparticles increased with the increased concentration of chloramphenicol used to coat the material (Tables S1 and S2). Results describing the percentage of chloramphenicol released over time (Fig. 1S) were converted to present the drug concentration expressed in mg per cm^2^ of surface area per time unit (mg/cm^2^)/h (Fig. 4).

The analysis of variance (Tables S3 and S4) shows that the release profiles differ from each other (*p* < 0.05) in terms of the chloramphenicol release rate (Fig. S2). It can be seen that there is a significant difference (*p* < 0.05) between the commercial lipophilic detreomycin (line with black triangles) and other hydrogel formulations prepared on the basis of Carbopol (Figs. 4 and S2). At the 5th minute of the test, there is a difference in the percentage of the active substance released from the tested formulations. Carbopol-based formulations achieve a higher maximum chloramphenicol concentration than the reference form (Detreomycin-2%) due to the complete difference in the medium used (hydrophilic carbopol-based substrate in the tested formulations vs lipophilic vaseline-based substrate in the commercial Detreomycin). The use of a hydrogel formulation provides a faster release of the active ingredient from the formulation, which can translate into a therapeutic effect. Both formulations based on nanocarriers (AuNPs and SiNPs) release the therapeutic substance significantly more efficiently than the commercial deteromecin-2% (*p* < 0.05), and the formulation containing chloramphenicol suspended in Carbopol gel (C/0.5%ClPh)-from the 5th minute of the conducted study. The advantage due to the higher efficiency of API release from hydrogels prepared with nanocarriers over the commercial formulation of detreomycin is, therefore, unquestionable.

The comparison of the formulations containing nanoparticles (AuNPs and SiNPs) coated with chloramphenicol shows a higher amount of drug released from the formulation based on the AuNPs carrier than from the formulation using SiNPs. The results strongly correlate with the obtained rheological data (Table 2). It should be noted that the C/AuNPs-0.5%ClPh formulation was characterised by larger polymer network mesh sizes and lower polymer network entanglement density, compared to the C/SiNPs-0.5%ClPh formulation, which translates into more efficient release of chloramphenicol from the C/AuNPs-based formulation (Table 2, item 2 and 4). It should be added that AuNPs are characterised by higher API release efficiencies in the tested formulations than SiNPs, which are related to the viscoelastic behaviour of the tested samples (Table 2), as well as the character of interactions of the polymer matrix with the nanoparticles. It should be noted that AuNPs demonstrate hydrophobic qualities as they do not form hydrogen bonds with hydrogel macromolecules, in opposition to SiNPs which have free -OH groups on the surface, capable of forming hydrogen bonds with the hydrogel. The presence of hydrogen bonds results in a slower release of the active ingredient from the tested formulations during the test (Figs. 4 and S2).

### Antimicrobial susceptibility testing

#### Disk diffusion test

**Fig. 5:**
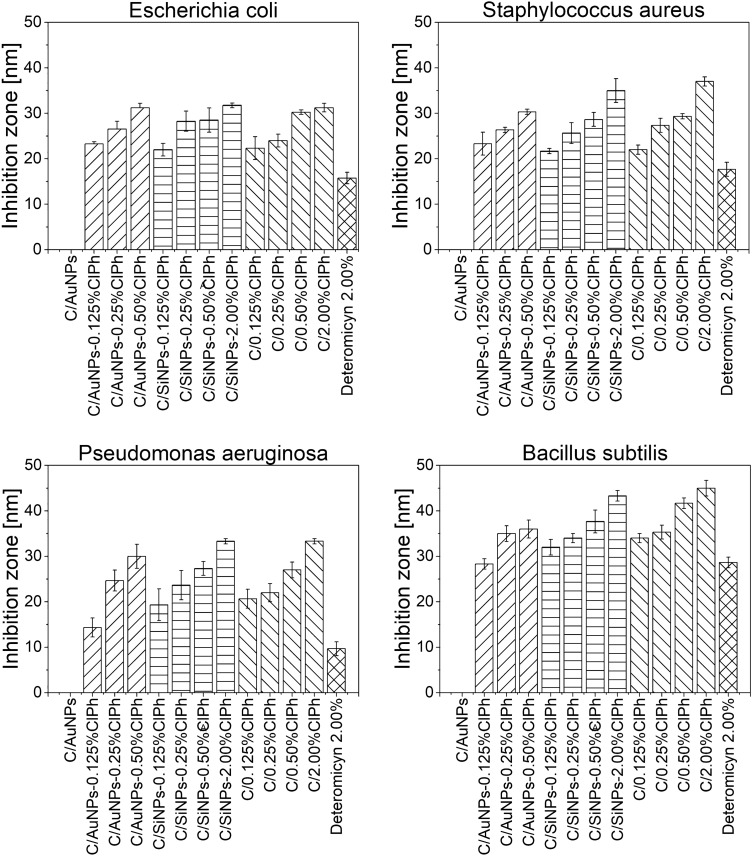
Mean inhibition zone diameters for tested microorganism. The test was performed using the Mueller Hinton Agar. Plates were struck to form a bacterial lawn. The diameter of the disks used in the experiment was 6 mm. Each disc was loaded with (12 ± 0.1) mg of the tested gel. Plates were inverted and incubated at (36 ± 1)°C for 24 h in a humid (~ 90%) atmosphere. Thereupon, the diameter of clear zones was measured. A Fisher’s LSD test was used to compare microbial inhibition zone values (Tables S6 and S8–S11). All calculated results are presented in supplementary materials

The bacteriostatic activity of selected hydrogel-based samples and the reference sample (Deteromecin-2%) was established by determining the growth inhibition zones (Table S5) using five reference strains with decreasing sensitivity to chloramphenicol (*Staphylococcus aureus* ATTC 6538P > *Escherichia coli* ATTC 8739 > *Bacillus subtilis* ATTC 6633 > *Pseudomonas aeruginosa* ATTC 27853 > *Candida albicans* ATTC 10231). Interestingly, carbopol-based formulations showed higher bacteriostatic activity, when compared to the commercially available detreomycin (Detreomycin-2.0%) (*p* < 0.05) (Fig. 5, Tables S6 and S8-S11). It has been shown that it is possible to significantly reduce the concentration of the antibiotic present in the formulation while maintaining and/or improving the bacteriostatic activity of chloramphenicol. A Fisher’s LSD test was used to compare microbial inhibition zone values (Tables S8-S11). All calculated results are presented in supplementary materials. In all analyses and statistical tests performed, a significance level of *α* = 0.05 was adopted (Table S6). Also, for better visualization, photos of the microbial inhibition zone for three reference strains *B. subtilus, E. coli* and *S. aureus* were presented in supplementary materials (Table S12).

According to the literature data [42, 45, 46], chloramphenicol was expected to have a strong bacteriostatic effect against the *E. coli* and *S. aureus* strains, a weaker effect against the *B. subtilus* strain (although the effect is strain-dependent; there are resistant as well as ClPh-sensitive strains), very weak against the *P. aruginosa* strain, and no fungistatic effect against the *C. albicans* fungus. The experimentally obtained results are presented in Table S5. Thus, the sensitivity of the strains experimentally determined against the commercial preparation (Table S5, item 13 and Table S12, item 13) was as follows: *Bacillus subtilis* ATTC 6633 > *Staphylococcus aureus* ATTC 6538P > *Escherichia coli* ATTC 8739 > *Pseudomonas aeruginosa* ATTC 27853 > *Candida albicans* ATTC 10231.

In terms of the experimentally determined sensitivity of microorganisms to ChPl, the strain with the highest sensitivity was *B. subtilis*. What is more, C/AuNPs-0.125% displayed an identical bacteriostatic activity as the reference Detreomycin-2% formulation (*p* = 0.43) (Table S12, items 2 and 13). It was also proven that the concentration of the antibiotic does not have a significant effect on the obtained values of inhibition zones of the microorganism because as the concentrations of the antibiotic increased, statistically significant differences in the obtained results were not always observed (Table S8, results marked in black, and Table S12 column *B. subtilis*). Thus, the substrate’s hydrophilic nature is decisive for the obtained values of microorganism inhibition zones, as each of the tested carbopol formulations showed higher activity than the commercial lipophilic Detreomycin-2% (Table S8, probability results for Detreomycin-2%). Along with the lower sensitivity of strains to ClPh, the influence of both the type of nanoparticles used and the rheological properties of the test material, such as the character of the polymer network, the crosslinking density of the polymer network or the composite viscosity on the results is noticeable. The opportunity to reduce the concentration of the antibiotic while maintaining and/or even improving the bacteriostatic activity of detreomycin is also noticeable. The highest bacteriostatic activity against the *S. aureus* strain was a characteristic of the C/2%ClPh formulation, which showed statistically significant differences against every tested formulation (Table S9, item C/2%ClPh). Only 3 of the tested formulations achieved an inhibition zone of more than 30 mm: C/2%-ClPh, C/SiNPs-2%ClPh, and C/AuNPs-0.5%ClPh (Tables S5 and S12, items 4, 12 and 13 for *S.aureus*). Towards the *E. coli* reference strain, the observed values of inhibition zones are almost identical to those obtained for *S.aureus* (Tables S5 and S12 results for *E.coli* and *S.aureus*).

On the other hand, the C/AuNPs-0.5%, C/SiNPs-2%ClPh and C/2%ClPh formulations turned out to be the most effective hydrogels among C/AuNPs-0.5%, C/SiNPs-2%ClPh and C/2%ClPh due to the observed inhibition zones. (Tables S5 and S12, items 4, 8, 12, *E.coli*). When compared to commercially available detreomycin (Tables S5 and S12, item 13), an almost twice-fold increase in the inhibition zone was observed in each of the aforementioned cases. The last reference strain tested was *P. aeruginosa*, with the lowest sensitivity to chloramphenicol of all the bacterial strains tested. The results in terms of inhibition zone values have documented the superiority of AuNPs over SiNPs in the range of the applied ClPh concentrations from 0.125% to 0.50% (Table S5, item 2 vs. 5, 3 vs. 6 and 4 vs. 7 for *P. aruginosa*). For this strain, the C/AuNPs-0.5%, C/SiNPs-2%ClPh and C/2%ClPh formulations were characterised by the highest bacteriostatic activity (Table S11), which reached inhibition zone values three times higher than the commercial preparation (Detreomycin-2%) (Table S5, item 4, 8, 12 and 13 for *P. aruginosa*). In addition, a strain without a chloramphenicol handle point, *C. albicans*, was also tested to assess whether the addition of nanogold could induce a fungicidal and/or fungistatic effect—unfortunately, too low a concentration of AuNPs failed to produce an antifungal effect (Table S5, item 1–13 for *C. albicans*).

Thus, both the substrate change to a more hydrophilic one and the addition of AuNPs or SiNPs make it possible to significantly reduce the concentration and improve the bacteriostatic activity of chloramphenicol in referrence to the commercially available lipophilic form of detreomycin. The difference in inhibition zones is as big as 23 mm (Table S5, items 4, 8 and 13 for *P. aeruginosa*), which confirms that both the nature of the substrate and the degree of crosslinking of the polymer network affects not only the rate of chloramphenicol release but also translates into the bacteriostatic activity of the tested API. Interestingly, it was found that the higher η* values observed for C/AuNPs-ClPh hydrogels (AuNPs), compared to C/SiNPs-ClPh hydrogels (SiNPs), translate into the higher bacteriostatic activity of gels prepared with AuNPs relative to SiNPs, which is particularly evident for strains with low sensitivity to chloramphenicol. It is also important to note the drug carrier weight ratios used, which are different for the released formulations containing 0.5%ClPh: SiNPs (2:1), AuNPs (152:1), which translates into the strength of intermolecular interactions present in the carbopol hydrogel, which in turn determine the observed bacteriostatic activities of the tested formulations. Thus, by using nanoparticles, what can be improved is not only can patient safety against such a toxic drug as chloramphenicol, but also the bacteriostatic activity of the antibiotic.

The results show that it is possible to reduce the concentration of chloramphenicol fourfold in a formulation containing 0.50% chloramphenicol while maintaining identical bacteriostatic activity as in a commercially available formulation (Detreomycin-2%).

#### Bacterial growth kinetics

**Fig. 6:**
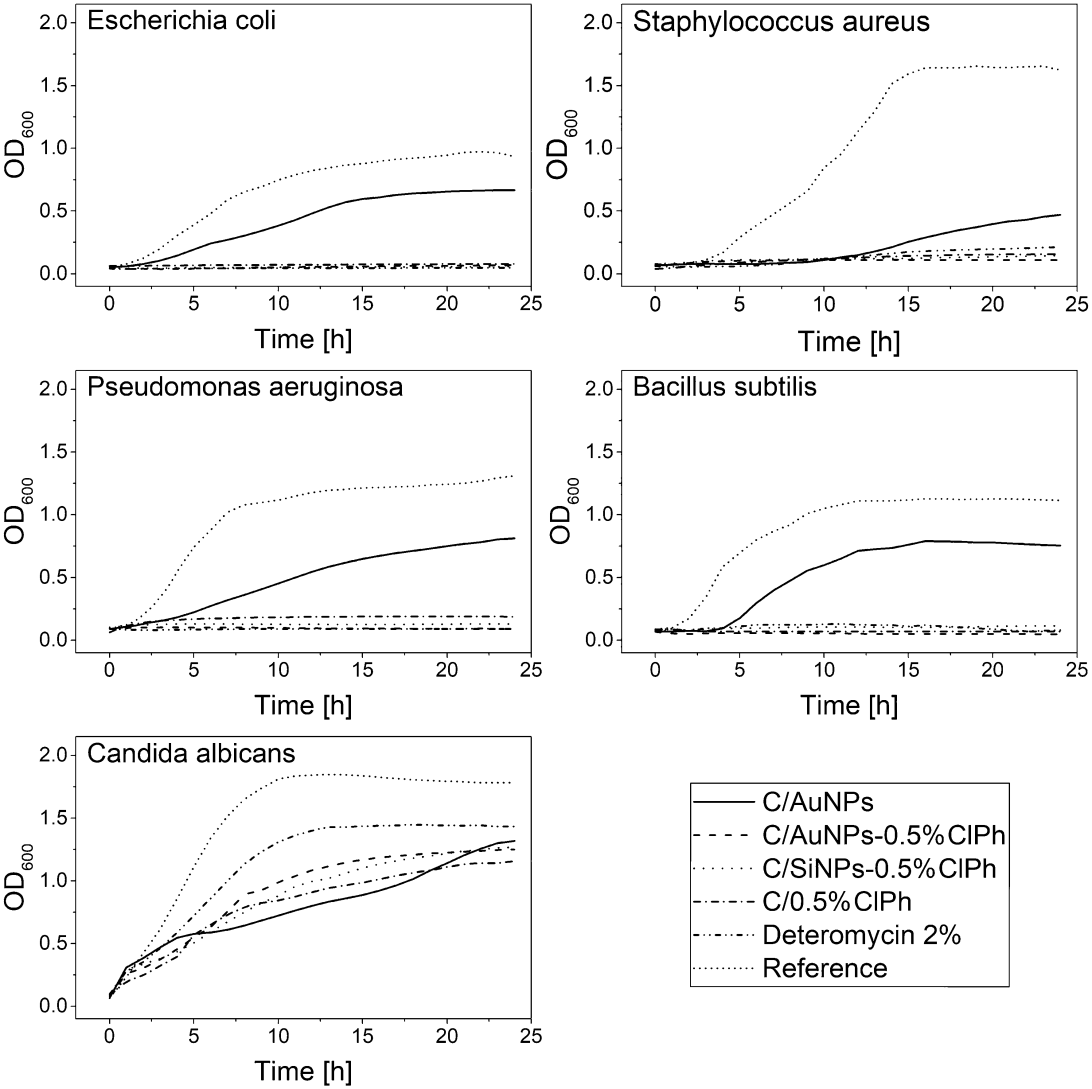
Microorganism growth kinetics tested for selected formulations. The test was performed by mixing 150 μL of inoculum and 450 μL of gel in 24-well polystyrene cell culture plates (NEST^®^Scientific). A mixture of 150 μL of inoculum and 450 μL PBS was used as a reference. Plates were incubated at (36 ± 1)°C for 24 h with an orbital shaking of 5-mm amplitude and frequency of 120 rpm in TECAN Spark^®^ 20 M. Every 60 min, the absorbance signal was read at 600 nm. A Fisher’s LSD test was used to compare OD600nm value of MH Broth (Tables S7 and S13–S17). All calculated results are presented in supplementary materials

A bacterial growth kinetics study was performed to verify the bacteriostatic effect of chloramphenicol and the possible effect of AuNPs and SiNPs on the growth of model strains. Each of the carbopol-based formulations tested shows statistically significant differences in inhibition of bacterial growth kinetics relative to the control formulation (no antibiotic added—dotted line, reference on Fig. 6) and no effect in inhibition of fungal growth (*C. albicans*). The addition of gold nanoparticles (C/AuNPs formulation, solid black line) slightly inhibits the bacterial growth kinetics (Fig. 6 and Fig. S3 (presented in the faintly visible area)). No significant statistical differences were observed between the reference drug (Detreomycin-2%) and the tested formulations in terms of inhibition kinetics of bacterial growth (*p* > 0.05) (Tables S7 and S13-S17). All calculated results of Fisher’s LSD test for OD600nm value of MH Broth are presented in supplementary materials.

For the reference strain with the highest sensitivity to chloramphenicol, i.e*. B. subtilis*, no statistically significant differences were observed for all hydrogel formulations against the lipophilic reference drug (Detreomycin-2%) *p* > 0.05. Significantly, there is a noticeable difference between C/AuNPs-0.5%ClPh and the C/SiNPs-0.125%ClPh, C/SiNPs-2.0%ClPh and C/2.0%ClPh formulations, in favour of formulations using AuNPs, which better limit the growth kinetics of the reference strain (Table S13, item AuNPs, result marked in red colour). Against *S. aureus*, no statistically significant differences in limiting the growth kinetics of the microorganism were observed between the C/SiNPs-0.125% and C/0.50%ClPh formulations (*p* > 0.05). Thus, the addition of SiNPs does not always change the kinetics of bacterial growth (Table S14). Against *E. coli*, there were no statistically significant differences between the reference drug (Detreomycin-2%) and the tested hydrogel formulations in terms of microbial growth inhibition kinetics (Table S15). For the strain with the lowest sensitivity to the tested antibiotic, i.e. *P. aeruginosa,* it could be observed that gold nanoparticles (AuNPs) significantly affect the inhibition of bacterial growth kinetics, as there is a statistically significant difference between the control formulation (Detreomycin-2%) and the C/AuNPs formulation without the antibiotic (*p* < 0.05) (Table S16). Thus, it can be assumed that for strains that are not very sensitive to chloramphenicol, adding AuNPs significantly affects the growth kinetics of microorganisms. In terms of the attempt to inhibit the growth kinetics of fungi (*C. albicans*), the results confirm the previous observations that AuNPs without antibiotic addition do not show statistically significant differences with the other tested formulations, so the addition of nanogold is insufficient to achieve a fungicidal effect (Table S17). In summary, all tested hydrogel formulations affect and limit bacterial growth kinetics, while at the same time, the addition of nanogold shows no fungicidal effect. Given the possibility of limiting microbial growth by using gold nanoparticles (AuNPs) without coating them with ClPh, adding these nanoparticles seems as reasonable and preferable as SiNPs.

Thus, it is the C/AuNPs-0.5%ClPh formulation is the most promising, due to the observed release profile as well as the obtained values of the inhibition zones of the tested microorganisms and the ability of AuNPs to inhibit microbial growth kinetics.

## Conclusion

The study proved that the technological modification involving the coating of chloramphenicol on carriers (SiNPs and AuNPs) showed to be expedient and reasonable. The addition of nanogold and silica increased the bacteriostatic activity of the tested antibiotic and, in some cases, reduced the chloramphenicol concentration necessary to achieve a bacteriostatic effect. The preferred formulation, due to its release profile, as well as the described rheological properties and bacteriostatic activity, was the C/AuNPs-0.5%ClPh formulation. In addition, it has been shown that the mere change of medium (in this case to hydrogel), as well as the addition of nanocarriers (SiNPs or AuNPs), can modify the growth kinetics of microorganisms and affect the bacteriostatic activity of the test formulations. There is a need for further research and optimisation of the obtained formulations to confirm the obtained results. Nevertheless, the work indicates that it is possible to reduce the concentration of chloramphenicol by four times while maintaining its bacteriostatic activity, which can improve the patient’s safety profile while increasing the effectiveness of the therapy. The important notion is that the incorporation of gold into nanomaterials may be a premise that contributes to changing the registration indications of chloramphenicol.

## Supplementary Information

Below is the link to the electronic supplementary material.

Supplementary file1 (DOCX 1705 KB)

## Data Availability

All necessary data generated or analyzed during this study are included in this published article [and its supplementary information files]. Other required data (if needed) are available from the corresponding author upon reasonable request.

## Abbreviations

API: Active pharmaceutical ingredients
AuNPs: Gold nanoparticles
C/: Carbopol based formulation
ClPh: Chloramphenicol
Gʹ: Storage modulus
G": The loss modulus
LVE: Region for investigated samples
MIC: Minimum inhibitory concentrations
NPs: Nanoparticles
OD_600nm_: Optical density at 600 nm
SiNPs: Silica nanoparticles
ξ: Network mesh sizes
η*: Complex viscosity
ω: Oscillation frequency
