# Supplementary material for: Nanoparticles coated by chloramphenicol in hydrogels as a useful tool to increase the antibiotic release and antibacterial activity in dermal drug delivery
Source: Pharmacol Rep. 2023 Apr 11;75(3):657–70. doi: 10.1007/s43440-023-00482-4 (PMC10227170; doi:10.1007/s43440-023-00482-4)
Supplement: Supplementary file 1 — Supplementary file1 (DOCX 1705 KB) [file 43440_2023_482_MOESM1_ESM.docx]

Nanoparticles coated by chloramphenicol in hydrogels as a useful tool to increase the antibiotic release and antibacterial activity in dermal drug delivery

**Dawid Bursy^1^, Radosław Balwierz^2*^, Paweł Groch^1^, Paweł Biernat^1^, Adam Bryski^3^, Katarzyna Kasperkiewicz^4^, Wioletta Ochędzan – Siodłak^2^**

^1^ Department of Drug Forms Technology, Faculty of Pharmacy, Borowska St. 211, Wrocław Medical University, 50-556 Wrocław, Poland

^2^ Institute of Chemistry, University of Opole, Oleska St. 48, 45-052 Opole, Poland

^3^ Institute of Metallurgy and Materials Science, Polish Academy of Sciences, Reymonta St. 25, 30-059 Cracow, Poland

^4^ University of Silesia in Katowice, Faculty of Natural Sciences, Institute of Biology, Biotechnology and Environmental Protection, Jagiellońska St. 28, 40-032 Katowice, Poland

* Correspondence: radoslaw.balwierz@uni.opole.pl

| **Table of contents** | **p.** |
| --- | --- |
| Methodology supplement | 2 |
| **Table S1.** Content of the active substance in a 0.5% carbpol gel as determined by the HPLC method | 4 |
| **Table S2**. Statistical summary of the particle size measurements (nm) for AuNPs and SiNPs obtained using the dynamic light scattering (DLS) method. | 4 |
| **Figure S1.** Q diagram for the values of the released percentage over time. | 4 |
| **Table S3.** Analysis of variance for tested hydrogels and commercial detreomycin | 5 |
| **Table S4.** Results of statistical analysis for released formulation | 5 |
| **Figure S2.** Comparison of chloramphenicol release rate according to Higuchi model | 5 |
| **Table S5.** Inhibition zone for tested formulation | 6 |
| **Table S6.** Results of main effect of ANOVA analysis for inhibition zones of tested formulations | 6 |
| **Table S7.** Results of main effect of ANOVA analysis for OD600 of tested formulations | 6 |
| **Table S8.** Results of Fisher’s LSD Test for *B. subtilis* inhibition zones of tested formulations | 7 |
| **Table S9.** Results of Fisher’s LSD Test for *S. aureus* inhibition zones of tested formulations | 8 |
| **Table S10.** Results of Fisher’s LSD Test for *E. coli* inhibition zones of tested formulations | 9 |
| **Table S11.** Results of Fisher’s LSD Test for *P. aruginosa* inhibition zones of tested formulations | 10 |
| **Table S12.** Selected photos of inhibitions zone for tested formulation | 11 |
| **Figure S3.** Microorganisms growth kinetics tested for selected formulations (the faintly visible area from figure 6) | 14 |
| **Table S13.** Results of Fisher’s LSD Test for *B. subtilis* OD600 of tested formulations | 15 |
| **Table S14.** Results of Fisher’s LSD Test for *S. aureus* OD600 of tested formulations | 16 |
| **Table S15.** Results of Fisher’s LSD Test for *E. coli* OD600 of tested formulations | 17 |
| **Table S16.** Results of Fisher’s LSD Test for *P. aruginosa* OD600 of tested formulations | 18 |
| **Table S17.** Results of Fisher’s LSD Test for *C.albicans* OD600 of tested formulations | 19 |

**Methodology supplement**

**Content of the chloramphenicol in the carbopol gel (chapter 2.6.)**

The analysis was carried out using an Agilent Infinity 1260 HPLC system equipped with a ThermoScientific column 4.6 × 150 mm, with a particle size of 5 µm and a C18 hypersil Gold bed. The mobile phase consisted of a mixture of acetonitrile and water in a 40:60 volume ratio. The measurement time for a single run was 6 minutes. The injection volume was 20 µL, and the thermostat temperature was set at 25°C. The sample was analyzed using a photodiode array detector with signal analysis at a detector wavelength of 278.4 nm.

The gel sample (100 mg) was placed in a 25 mL class A volumetric flask and filled with ethanol. The sample was stirred for 15 minutes using an ultrasonic cleaner and then refrigerated for 48 hours prior to measurement. The average retention time was 3.28 minutes. The selectivity of the method was confirmed by repeating the analysis with a gel containing silicon nanoparticles uncoated with chloramphenicol, which showed no signals in the retention time region for chloramphenicol. The precision of the method was confirmed by injecting the standard solution six times and obtaining a relative standard deviation of 1.95%.

**The release study of chloramphenicol (chapter 2.7.)**

The examination of the release of chloramphenicol from the nanocarriers were performed using an SR8PLUS pharmacopeial paddle dissolution apparatus with Enhancer cell vessels, an Agilent 850DS autosampler, and borate buffer as the acceptor medium. The study was conducted at 32°C with a constant stirring speed of 90 rpm for 6 hours, and a Cuprophane dialysis membrane was used to separate the donor from the acceptor. The thickness of the non-wetted membrane was 11.5 µm, and the molecular cut-off ratio was 10,000 Da. The study was conducted using two formulations obtained from respective syntheses of chloramphenicol-coated nanosilica particles (SiNPs-0.50%ClPh) and nanogold particles (AuNPs-0.50%ClPh) against two reference formulations prepared from chloramphenicol without a carrier (C/2.0%ClPh) and a commercial 2% chloramphenicol ointment produced by Chema-Elektroment (permit No. R/3286). Six units were tested for each formulation, and the autosampler sampled 2 mL of solution from each chamber at programmed measurement points. The study was conducted for eight measurement points: (I)—5 min, (II)—10 min, (III)—15 min, (IV)—30 min, (V)—60 min, (VI)—120 min, (VII)—240 min, and (VIII)—360 min. The acceptor solution samples collected by the autosampler were examined spectrophotometrically at λ = 278. The gel sample weights applied to the immersion chambers were verified to calculate each chamber's total active substance content.

**Table S1.** Content of the active substance in a 0.5% carbpol gel as determined by the HPLC method

| **Sample** | **Weight [mg]** | **CAP [mg]** | **Content [mg/100g]** | **Content relative to expected [%]** |
| --- | --- | --- | --- | --- |
| **C/0.5%ClPh** | 100.36 | 0.3955 | 492.6371 | 98.53% |
| **C/SiNPs-0.5%ClPh** | 101.15 | 0.3973 | 490.91 | 98.18% |
| **C/AuNPs-0.5%ClPh** | 107.80 | 0.3886 | 450.68 | 90.14% |
| C/0.5%ClPh – carbopol formulation based only on chloramphenicol (without the silica or nanogold carriers); C/SiNPs-0.5%ClPh - carbopol formulation based on SiNPs carrier and chloramphenicol; C/AuNPs-0.5%ClPh - carbopol formulation based on AuNPs carrier and chloramphenicol; CAP – capacity | | | | |

**Table S2**. Statistical summary of the particle size measurements (nm) for AuNPs and SiNPs obtained using the dynamic light scattering (DLS) method.

| **Material** | **Average [nm]** | **Std. Dev.** | **RSD** | **Minimum [nm]** | **Median [nm]** | **Maximum [nm]** |
| --- | --- | --- | --- | --- | --- | --- |
| **AuNPs-0%ClPh** | 6.012 | 1.812 | 30.13% | 2.448 | 6.1155 | 8.473 |
| **AuNPs-0.125%ClPh** | 9.420 | 2.350 | 24.95% | 4.415 | 9.6515 | 13.47 |
| **AuNPs-0.25%ClPh** | 127.3 | 25.6 | 20.15% | 30.79 | 130.55 | 158.3 |
| **AuNPs-0.5%ClPh** | 105.0 | 34.7 | 33.02% | 14.29 | 118.8 | 129.4 |
| **SiNPs-0%ClPh** | 107.71 | 21.97 | 20.39% | 62.78 | 112.75 | 131.0 |
| **SiNPs-0.125%ClPh** | 231.84 | 5.24 | 3.98% | 221.8 | 232.4 | 241.4 |
| **SiNPs-0.25%ClPh** | 260.0 | 77.8 | 29.9% | 168.7 | 257.3 | 440.4 |
| **SiNPs-0.5%ClPh** | 216.32 | 11.52 | 9.90% | 192.30 | 219.9 | 230.3 |
| AuNPs – gold nanoparticles; SiNPs – silica nanoparticles, ClPh - chloramphenicol | | | | | | |

**Figure S1.** Q diagram for the values of the released percentage over time.

C/0.5%ClPh – carbopol formulation based only on chloramphenicol (without the silica or nanogold carriers); C/SiNPs-0.5%ClPh - carbopol formulation based on SiNPs carrier and chloramphenicol; C/AuNPs-0.5%ClPh - carbopol formulation based on AuNPs carrier and chloramphenicol;

**Table S3.** Analysis of variance for tested tested hydrogels and commercial detreomycin

| Analysis of variance (SiNPs vs AuNPs Release).  Marked effects are significant with p < .05000 | | | | | | | | |
| --- | --- | --- | --- | --- | --- | --- | --- | --- |
| **variable** | **SS effect** | **df effect** | **MS effect** | **SS error** | **df**  **error** | **MS error** | **F** | **p** |
| K- Higuchi model constant [mg/cm^2^/h] | 0.1333 | 3 | 0.0444 | 0.0253 | 20 | 0.0013 | 35.1723 | 0.0000 |

**Table S4.** Results of statistical analysis for released formulation

| **Parameter: K Higuchi model** | **Formulation** | | | | **All Groups** |
| --- | --- | --- | --- | --- | --- |
|  | **C/0.5% ClPh** | **C/AuNPs -0.5%ClPh** | **C/SiNPs-0.5 ClPh** | **Detreomycin-2%** |  |
| Mean | 0.463 | 0.530 | 0.498 | 0.334 | 0.456 |
| Number | 6 | 6 | 6 | 6 | 24 |
| Std. Dev. | 0.048 | 0.023 | 0.010 | 0.047 | 0.083 |
| Std. Error | 0.019 | 0.009 | 0.004 | 0.019 | 0.017 |
| CV | 10% | 4% | 2% | 14% | 18% |
| Confidence level -0.95 | 0.413 | 0.506 | 0.488 | 0.285 | 0.421 |
| Confidence level +0.95 | 0.513 | 0.554 | 0.508 | 0.382 | 0.491 |

**Figure S2.** Comparison of chloramphenicol release rate according to Higuchi model.

C/0.5%ClPh – carbopol formulation based only on chloramphenicol (without the silica or nanogold carriers); C/SiNPs-0.5%ClPh - carbopol formulation based on SiNPs carrier and chloramphenicol; C/AuNPs-0.5%ClPh - carbopol formulation based on AuNPs carrier and chloramphenicol;

**Table S5.** Inhibition zone for tested formulation

| **No** | **Formulation** | **inhibition zone [mm]** | | | | | | | | | |
| --- | --- | --- | --- | --- | --- | --- | --- | --- | --- | --- | --- |
|  |  | ***E.coli*** | | ***S.aureus*** | | ***P.aeruginosa*** | | ***B.subtilis*** | | ***C.albicans*** | |
|  |  | **average** | **sd** | **average** | **sd** | **average** | **sd** | **average** | **sd** | **average** | **sd** |
| 1 | C/AuNPs | 0.0000 | 0.0000 | 0.0000 | 0.0000 | 0.0000 | 0.0000 | 0.0000 | 0.0000 | 0.0000 | 0.0000 |
| 2 | C/AuNPs-0.125%ClPh | 23.2500 | 0.5000 | 23.5000 | 2.0817 | 14.2500 | 1.7078 | 28.0000 | 1.1547 | 0.0000 | 0.0000 |
| 3 | C/AuNPs-0.25%ClPh | 26.5000 | 1.7321 | 26.2500 | 0.5000 | 24.7500 | 1.8930 | 35.5000 | 1.7321 | 0.0000 | 0.0000 |
| 4 | C/AuNPs-0.5%ClPh | 31.2500 | 0.9574 | 30.5000 | 0.5774 | 30.5000 | 2.3805 | 35.7500 | 1.7078 | 0.0000 | 0.0000 |
| 5 | C/SiNPs-0.125%ClPh | 22.0000 | 1.4142 | 21.7500 | 0.5000 | 19.5000 | 2.8868 | 32.0000 | 1.4142 | 0.0000 | 0.0000 |
| 6 | C/SiNPs-0.25%ClPh | 28.2500 | 2.2174 | 25.5000 | 1.9149 | 24.0000 | 2.7080 | 34.0000 | 0.8165 | 0.0000 | 0.0000 |
| 7 | C/SiNPs-0.5%ClPh | 28.5000 | 2.6458 | 28.5000 | 1.2910 | 27.2500 | 1.2583 | 37.7500 | 2.0616 | 0.0000 | 0.0000 |
| 8 | C/SiNPs-2.0%ClPh | 31.7500 | 0.5000 | 35.0000 | 2.1602 | 33.2500 | 0.5000 | 43.0000 | 1.1547 | 0.0000 | 0.0000 |
| 9 | C/0.125%ClPh | 22.5000 | 2.0817 | 22.0000 | 0.8165 | 20.7500 | 1.7078 | 33.7500 | 0.9574 | 0.0000 | 0.0000 |
| 10 | C/0.25%ClPh | 24.0000 | 1.4142 | 27.0000 | 1.4142 | 22.5000 | 1.9149 | 35.7500 | 1.5000 | 0.0000 | 0.0000 |
| 11 | C/0.5%ClPh | 27.7500 | 4.5000 | 29.5000 | 0.5774 | 26.5000 | 1.7321 | 42.0000 | 1.1547 | 0.0000 | 0.0000 |
| 12 | C/2.0%ClPh | 31.2500 | 0.9574 | 37.2500 | 0.9574 | 33.2500 | 0.5000 | 45.2500 | 1.5000 | 0.0000 | 0.0000 |
| 13 | Detreomycin 2.0% | 15.7500 | 1.2583 | 18.0000 | 1.4142 | 9.7500 | 1.2583 | 28.7500 | 0.9574 | 0.0000 | 0.0000 |
| C/ - carbopol based formulation; AuNPs – gold nanoparticles; SiNPs – silica nanoparticles, ClPh - chloramphenicol | | | | | | | | | | | |

**Table S6.** Results of main effect of ANOVA analysis for inhibition zones of tested formulations

| Effect | Multivariate Tests of Significance  Sigma-restricted parameterization Effective hypothesis decomposition  Marked effects are significant with p < .05000 | | | | | |
| --- | --- | --- | --- | --- | --- | --- |
|  | \| Test \| \| --- \| | \| Value \| \| --- \| | \| F \| \| --- \| | \| Effect df \| \| --- \| | \| Error df \| \| --- \| | \| p \| \| --- \| |
| \| Intercept \| \| --- \| | Wilks | 0.000519 | 17316.15 | 4 | 36.0000 | 0.00 |
| \| Formulation \| \| --- \| | Wilks | 0.000138 | 26.51 | 48 | 140.7144 | 0.00 |

**Table S7.** Results of main effect of ANOVA analysis for OD600 of tested formulations

| Effect | Multivariate Tests of Significance  Sigma-restricted parameterization Effective hypothesis decomposition  Marked effects are significant with p < .05000 | | | | | |
| --- | --- | --- | --- | --- | --- | --- |
|  | \| Test \| \| --- \| | \| Value \| \| --- \| | \| F \| \| --- \| | \| Effect df \| \| --- \| | \| Error df \| \| --- \| | \| p \| \| --- \| |
| \| Intercept \| \| --- \| | Wilks | 0,099166 | 603,1834 | 5 | 332,000 | 0,00 |
| \| Formulation \| \| --- \| | Wilks | 0,010264 | 39,5700 | 65 | 1572,914 | 0,00 |

**Table S8.** Results of Fisher’s LSD Test for *B. subtilis* inhibition zones of tested formulations

| No. g | LSD Test; variable B.subtilis inhibition zone [mm]  Probabilities for Post Hoc Tests  Error: Between MS = 1.7821. df = 39.000 | | | | | | | | | | | | | |
| --- | --- | --- | --- | --- | --- | --- | --- | --- | --- | --- | --- | --- | --- | --- |
|  | Formulation | C/AuNPs | C/AuNPs-0.125%ClPh | C/AuNPs-0.25%ClPh | C/AuNPs-0.5%ClPh | C/SiNPs-0.125%ClPh | C/SiNPs-0.25%ClPh | C/SiNPs-0.5%ClPh | C/SiNPs-2.0%ClPh | C/0.125%ClPh | C/0.25%ClPh | C/0.5%ClPh | C/2.0%ClPh | Detreomycin 2.0% |
| 1 | C/AuNPs |  | 0.0000 | 0.0000 | 0.0000 | 0.0000 | 0.0000 | 0.0000 | 0.0000 | 0.0000 | 0.0000 | 0.0000 | 0.0000 | 0.0000 |
| 2 | C/AuNPs-0.125%ClPh | 0.000 |  | 0.0000 | 0.0000 | 0.0001 | 0.0000 | 0.0000 | 0.0000 | 0.0000 | 0.0000 | 0.0000 | 0.0000 | 0.4317 |
| 3 | C/AuNPs-0.25%ClPh | 0.000 | 0.0000 |  | 0.7925 | 0.0006 | 0.1201 | 0.0221 | 0.0000 | 0.0713 | 0.7925 | 0.0000 | 0.0000 | 0.0000 |
| 4 | C/AuNPs-0.5%ClPh | 0.000 | 0.0000 | 0.7925 |  | 0.0003 | 0.0713 | 0.0405 | 0.0000 | 0.0405 | 1.0000 | 0.0000 | 0.0000 | 0.0000 |
| 5 | C/SiNPs-0.125%ClPh | 0.000 | 0.0001 | 0.0006 | 0.0003 |  | 0.0405 | 0.0000 | 0.0000 | 0.0713 | 0.0003 | 0.0000 | 0.0000 | 0.0014 |
| 6 | C/SiNPs-0.25%ClPh | 0.000 | 0.0000 | 0.1201 | 0.0713 | 0.0405 |  | 0.0003 | 0.0000 | 0.7925 | 0.0713 | 0.0000 | 0.0000 | 0.0000 |
| 7 | C/SiNPs-0.5%ClPh | 0.000 | 0.0000 | 0.0221 | 0.0405 | 0.0000 | 0.0003 |  | 0.0000 | 0.0001 | 0.0405 | 0.0001 | 0.0000 | 0.0000 |
| 8 | C/SiNPs-2.0%ClPh | 0.000 | 0.0000 | 0.0000 | 0.0000 | 0.0000 | 0.0000 | 0.0000 |  | 0.0000 | 0.0000 | 0.2959 | 0.0221 | 0.0000 |
| 9 | C/0.125%  ClPh | 0.000 | 0.0000 | 0.0713 | 0.0405 | 0.0713 | 0.7925 | 0.0001 | 0.0000 |  | 0.0405 | 0.0000 | 0.0000 | 0.0000 |
| 10 | C/0.25%ClPh | 0.000 | 0.0000 | 0.7925 | 1.0000 | 0.0003 | 0.0713 | 0.0405 | 0.0000 | 0.0405 |  | 0.0000 | 0.0000 | 0.0000 |
| 11 | C/0.5%ClPh | 0.000 | 0.0000 | 0.0000 | 0.0000 | 0.0000 | 0.0000 | 0.0001 | 0.2959 | 0.0000 | 0.0000 |  | 0.0014 | 0.0000 |
| 12 | C/2.0%ClPh | 0.000 | 0.0000 | 0.0000 | 0.0000 | 0.0000 | 0.0000 | 0.0000 | 0.0221 | 0.0000 | 0.0000 | 0.0014 |  | 0.0000 |
| 13 | Detreomycin 2.0% | 0.000 | 0.4317 | 0.0000 | 0.0000 | 0.0014 | 0.0000 | 0.0000 | 0.0000 | 0.0000 | 0.0000 | 0.0000 | 0.0000 |  |

C/ - carbopol based formulation; AuNPs – gold nanoparticles; SiNPs – silica nanoparticles, ClPh - chloramphenicol

**Table S9.** Results of Fisher’s LSD Test for *S. aureus* inhibition zones of tested formulations

| No. g | LSD Test; variable S.aureus inhibition zone [mm]  Probabilities for Post Hoc Tests  Error: Between MS = 1.6218. df = 39.000 | | | | | | | | | | | | | |
| --- | --- | --- | --- | --- | --- | --- | --- | --- | --- | --- | --- | --- | --- | --- |
|  | Formulation | C/AuNPs | C/AuNPs-0.125%ClPh | C/AuNPs-0.25%ClPh | C/AuNPs-0.5%ClPh | C/SiNPs-0.125%ClPh | C/SiNPs-0.25%ClPh | C/SiNPs-0.5%ClPh | C/SiNPs-2.0%ClPh | C/0.125%ClPh | C/0.25%ClPh | C/0.5%ClPh | C/2.0%ClPh | Detreomycin 2.0% |
| 1 | C/AuNPs |  | 0.0000 | 0.0000 | 0.0000 | 0.0000 | 0.0000 | 0.0000 | 0.0000 | 0.0000 | 0.0000 | 0.0000 | 0.0000 | 0.0000 |
| 2 | C/AuNPs-0.125%ClPh | 0.000 |  | 0.0041 | 0.0000 | 0.0592 | 0.0322 | 0.0000 | 0.0000 | 0.1038 | 0.0004 | 0.0000 | 0.0000 | 0.0000 |
| 3 | C/AuNPs-0.25%ClPh | 0.000 | 0.0041 |  | 0.0000 | 0.0000 | 0.4100 | 0.0168 | 0.0000 | 0.0000 | 0.4100 | 0.0009 | 0.0000 | 0.0000 |
| 4 | C/AuNPs-0.5%ClPh | 0.000 | 0.0000 | 0.0000 |  | 0.0000 | 0.0000 | 0.0322 | 0.0000 | 0.0000 | 0.0004 | 0.2736 | 0.0000 | 0.0000 |
| 5 | C/SiNPs-0.125%ClPh | 0.000 | 0.0592 | 0.0000 | 0.0000 |  | 0.0002 | 0.0000 | 0.0000 | 0.7828 | 0.0000 | 0.0000 | 0.0000 | 0.0002 |
| 6 | C/SiNPs-0.25%ClPh | 0.000 | 0.0322 | 0.4100 | 0.0000 | 0.0002 |  | 0.0019 | 0.0000 | 0.0004 | 0.1038 | 0.0001 | 0.0000 | 0.0000 |
| 7 | C/SiNPs-0.5%ClPh | 0.000 | 0.0000 | 0.0168 | 0.0322 | 0.0000 | 0.0019 |  | 0.0000 | 0.0000 | 0.1038 | 0.2736 | 0.0000 | 0.0000 |
| 8 | C/SiNPs-2.0%ClPh | 0.000 | 0.0000 | 0.0000 | 0.0000 | 0.0000 | 0.0000 | 0.0000 |  | 0.0000 | 0.0000 | 0.0000 | 0.0168 | 0.0000 |
| 9 | C/0.125%  ClPh | 0.000 | 0.1038 | 0.0000 | 0.0000 | 0.7828 | 0.0004 | 0.0000 | 0.0000 |  | 0.0000 | 0.0000 | 0.0000 | 0.0001 |
| 10 | C/0.25%ClPh | 0.000 | 0.0004 | 0.4100 | 0.0004 | 0.0000 | 0.1038 | 0.1038 | 0.0000 | 0.0000 |  | 0.0084 | 0.0000 | 0.0000 |
| 11 | C/0.5%ClPh | 0.000 | 0.0000 | 0.0009 | 0.2736 | 0.0000 | 0.0001 | 0.2736 | 0.0000 | 0.0000 | 0.0084 |  | 0.0000 | 0.0000 |
| 12 | C/2.0%ClPh | 0.000 | 0.0000 | 0.0000 | 0.0000 | 0.0000 | 0.0000 | 0.0000 | 0.0168 | 0.0000 | 0.0000 | 0.0000 |  | 0.0000 |
| 13 | Detreomycin 2.0% | 0.000 | 0.0000 | 0.0000 | 0.0000 | 0.0002 | 0.0000 | 0.0000 | 0.0000 | 0.0001 | 0.0000 | 0.0000 | 0.0000 |  |

C/ - carbopol based formulation; AuNPs – gold nanoparticles; SiNPs – silica nanoparticles, ClPh - chloramphenicol

**Table S10.** Results of Fisher’s LSD Test for *E. coli* inhibition zones of tested formulations

| No. g | LSD Test; variable E. coli inhibition zone [mm] Probabilities for Post Hoc Tests  Error: Between MS = 3.6474. df = 39.000 | | | | | | | | | | | | | |
| --- | --- | --- | --- | --- | --- | --- | --- | --- | --- | --- | --- | --- | --- | --- |
|  | Formulation | C/AuNPs | C/AuNPs-0.125%ClPh | C/AuNPs-0.25%ClPh | C/AuNPs-0.5%ClPh | C/SiNPs-0.125%ClPh | C/SiNPs-0.25%ClPh | C/SiNPs-0.5%ClPh | C/SiNPs-2.0%ClPh | C/0.125%ClPh | C/0.25%ClPh | C/0.5%ClPh | C/2.0%ClPh | Detreomycin 2.0% |
| 1 | C/AuNPs |  | 0.0000 | 0.0000 | 0.0000 | 0.0000 | 0.0000 | 0.0000 | 0.0000 | 0.0000 | 0.0000 | 0.0000 | 0.0000 | 0.0000 |
| 2 | C/AuNPs-0.125%ClPh | 0.000 |  | 0.0209 | 0.0000 | 0.3603 | 0.0007 | 0.0004 | 0.0000 | 0.5818 | 0.5818 | 0.0019 | 0.0000 | 0.0000 |
| 3 | C/AuNPs-0.25%ClPh | 0.000 | 0.0209 |  | 0.0011 | 0.0019 | 0.2026 | 0.1466 | 0.0004 | 0.0052 | 0.0717 | 0.3603 | 0.0011 | 0.0000 |
| 4 | C/AuNPs-0.5%ClPh | 0.000 | 0.0000 | 0.0011 |  | 0.0000 | 0.0322 | 0.0485 | 0.7132 | 0.0000 | 0.0000 | 0.0134 | 1.0000 | 0.0000 |
| 5 | C/SiNPs-0.125%ClPh | 0.000 | 0.3603 | 0.0019 | 0.0000 |  | 0.0000 | 0.0000 | 0.0000 | 0.7132 | 0.1466 | 0.0001 | 0.0000 | 0.0000 |
| 6 | C/SiNPs-0.25%ClPh | 0.000 | 0.0007 | 0.2026 | 0.0322 | 0.0000 |  | 0.8541 | 0.0134 | 0.0001 | 0.0032 | 0.7132 | 0.0322 | 0.0000 |
| 7 | C/SiNPs-0.5%ClPh | 0.000 | 0.0004 | 0.1466 | 0.0485 | 0.0000 | 0.8541 |  | 0.0209 | 0.0001 | 0.0019 | 0.5818 | 0.0485 | 0.0000 |
| 8 | C/SiNPs-2.0%ClPh | 0.000 | 0.0000 | 0.0004 | 0.7132 | 0.0000 | 0.0134 | 0.0209 |  | 0.0000 | 0.0000 | 0.0052 | 0.7132 | 0.0000 |
| 9 | C/0.125%  ClPh | 0.000 | 0.5818 | 0.0052 | 0.0000 | 0.7132 | 0.0001 | 0.0001 | 0.0000 |  | 0.2735 | 0.0004 | 0.0000 | 0.0000 |
| 10 | C/0.25%ClPh | 0.000 | 0.5818 | 0.0717 | 0.0000 | 0.1466 | 0.0032 | 0.0019 | 0.0000 | 0.2735 |  | 0.0084 | 0.0000 | 0.0000 |
| 11 | C/0.5%ClPh | 0.000 | 0.0019 | 0.3603 | 0.0134 | 0.0001 | 0.7132 | 0.5818 | 0.0052 | 0.0004 | 0.0084 |  | 0.0134 | 0.0000 |
| 12 | C/2.0%ClPh | 0.000 | 0.0000 | 0.0011 | 1.0000 | 0.0000 | 0.0322 | 0.0485 | 0.7132 | 0.0000 | 0.0000 | 0.0134 |  | 0.0000 |
| 13 | Detreomycin 2.0% | 0.000 | 0.0000 | 0.0000 | 0.0000 | 0.0000 | 0.0000 | 0.0000 | 0.0000 | 0.0000 | 0.0000 | 0.0000 | 0.0000 |  |

C/ - carbopol based formulation; AuNPs – gold nanoparticles; SiNPs – silica nanoparticles, ClPh - chloramphenicol

**Table S11.** Results of Fisher’s LSD Test for *P. aeruginosa* inhibition zones of tested formulations

| No. g | LSD Test; variable.aeruginosa inhibition zone [mm]  Probabilities for Post Hoc Tests  Error: Between MS = 3.1603. df = 39.000 | | | | | | | | | | | | | |
| --- | --- | --- | --- | --- | --- | --- | --- | --- | --- | --- | --- | --- | --- | --- |
|  | Formulation | C/AuNPs | C/AuNPs-0.125%ClPh | C/AuNPs-0.25%ClPh | C/AuNPs-0.5%ClPh | C/SiNPs-0.125%ClPh | C/SiNPs-0.25%ClPh | C/SiNPs-0.5%ClPh | C/SiNPs-2.0%ClPh | C/0.125%ClPh | C/0.25%ClPh | C/0.5%ClPh | C/2.0%ClPh | Detreomycin 2.0% |
| 1 | C/AuNPs |  | 0.0000 | 0.0000 | 0.0000 | 0.0000 | 0.0000 | 0.0000 | 0.0000 | 0.0000 | 0.0000 | 0.0000 | 0.0000 | 0.0000 |
| 2 | C/AuNPs-0.125%ClPh | 0.0000 |  | 0.0000 | 0.0000 | 0.0002 | 0.0000 | 0.0000 | 0.0000 | 0.0000 | 0.0000 | 0.0000 | 0.0000 | 0.0009 |
| 3 | C/AuNPs-0.25%ClPh | 0.0000 | 0.0000 |  | 0.0000 | 0.0002 | 0.5542 | 0.0538 | 0.0000 | 0.0029 | 0.0812 | 0.1718 | 0.0000 | 0.0000 |
| 4 | C/AuNPs-0.5%ClPh | 0.0000 | 0.0000 | 0.0000 |  | 0.0000 | 0.0000 | 0.0136 | 0.0348 | 0.0000 | 0.0000 | 0.0029 | 0.0348 | 0.0000 |
| 5 | C/SiNPs-0.125%ClPh | 0.0000 | 0.0002 | 0.0002 | 0.0000 |  | 0.0009 | 0.0000 | 0.0000 | 0.3262 | 0.0220 | 0.0000 | 0.0000 | 0.0000 |
| 6 | C/SiNPs-0.25%ClPh | 0.0000 | 0.0000 | 0.5542 | 0.0000 | 0.0009 |  | 0.0136 | 0.0000 | 0.0136 | 0.2400 | 0.0538 | 0.0000 | 0.0000 |
| 7 | C/SiNPs-0.5%ClPh | 0.0000 | 0.0000 | 0.0538 | 0.0136 | 0.0000 | 0.0136 |  | 0.0000 | 0.0000 | 0.0005 | 0.5542 | 0.0000 | 0.0000 |
| 8 | C/SiNPs-2.0%ClPh | 0.0000 | 0.0000 | 0.0000 | 0.0348 | 0.0000 | 0.0000 | 0.0000 |  | 0.0000 | 0.0000 | 0.0000 | 1.0000 | 0.0000 |
| 9 | C/0.125%  ClPh | 0.0000 | 0.0000 | 0.0029 | 0.0000 | 0.3262 | 0.0136 | 0.0000 | 0.0000 |  | 0.1718 | 0.0000 | 0.0000 | 0.0000 |
| 10 | C/0.25%ClPh | 0.0000 | 0.0000 | 0.0812 | 0.0000 | 0.0220 | 0.2400 | 0.0005 | 0.0000 | 0.1718 |  | 0.0029 | 0.0000 | 0.0000 |
| 11 | C/0.5%ClPh | 0.0000 | 0.0000 | 0.1718 | 0.0029 | 0.0000 | 0.0538 | 0.5542 | 0.0000 | 0.0000 | 0.0029 |  | 0.0000 | 0.0000 |
| 12 | C/2.0%ClPh | 0.0000 | 0.0000 | 0.0000 | 0.0348 | 0.0000 | 0.0000 | 0.0000 | 1.0000 | 0.0000 | 0.0000 | 0.0000 |  | 0.0000 |
| 13 | Detreomycin 2.0% | 0.0000 | 0.0009 | 0.0000 | 0.0000 | 0.0000 | 0.0000 | 0.0000 | 0.0000 | 0.0000 | 0.0000 | 0.0000 | 0.0000 |  |

C/ - carbopol based formulation; AuNPs – gold nanoparticles; SiNPs – silica nanoparticles, ClPh - chloramphenicol

**Table S12.** Selected photos of inhibitions zone for tested formulation

| **No** | **Formulation** | **inhibition zone photo** | | | | |
| --- | --- | --- | --- | --- | --- | --- |
|  |  | ***B. subtilis*** | ***S. aureus*** | ***E. coli*** | ***P. aeruginosa*** | ***C. albicans*** |
| 1 | C/AuNPs | 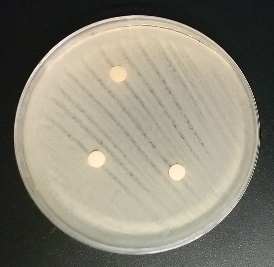 | 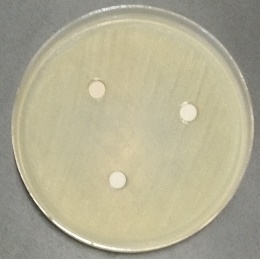 | 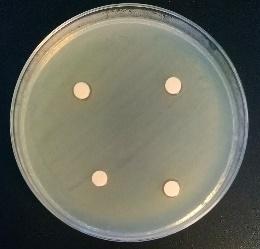 | 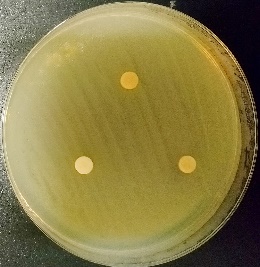 | 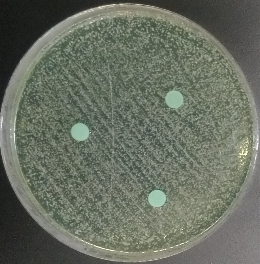 |
| 2 | C/AuNPs-0.125%ClPh | 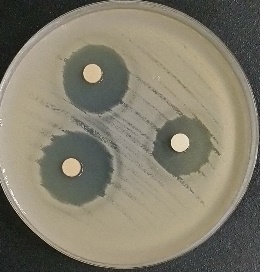 | 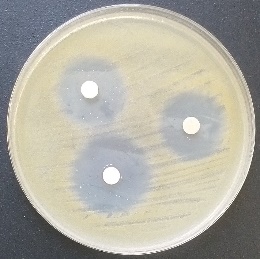 | 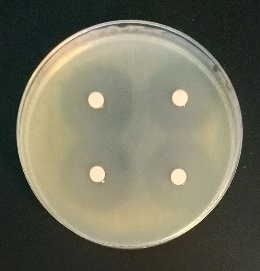 |  |  |
| 3 | C/AuNPs-0.25%ClPh | 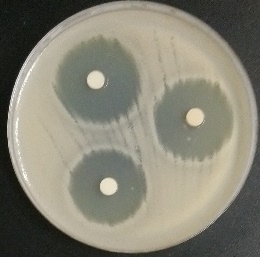 | 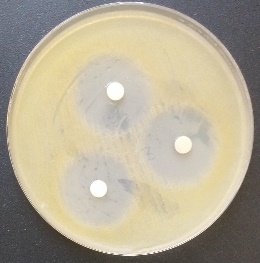 | 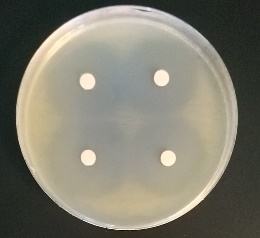 |  |  |
| 4 | C/AuNPs-0.5%ClPh | 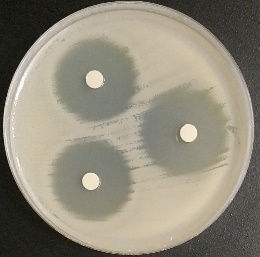 | 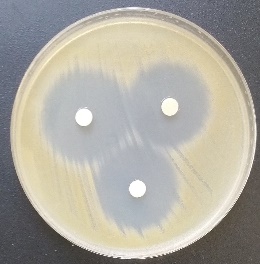 | 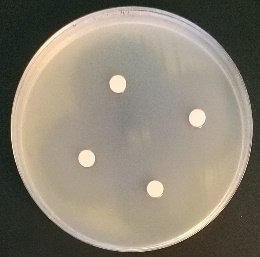 |  |  |
| 5 | C/SiNPs-0.125%ClPh | 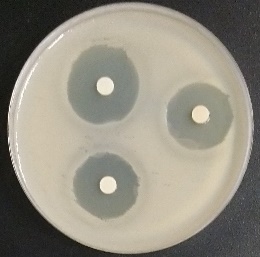 | 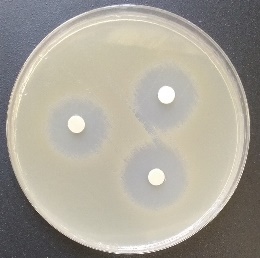 | 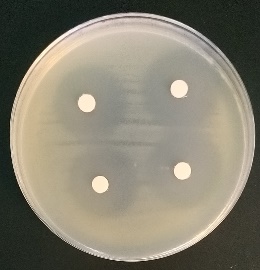 |  |  |
| 6 | C/SiNPs-0.25%ClPh | 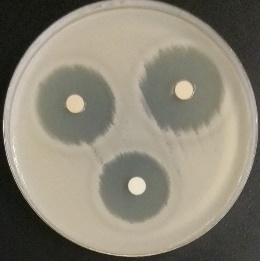 | 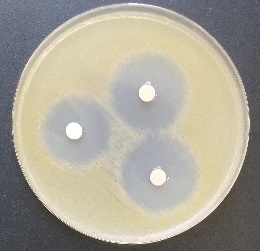 | 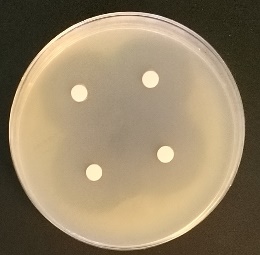 |  |  |
| 7 | C/SiNPs-0.5%ClPh | 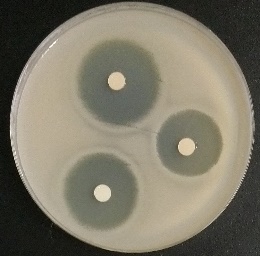 | 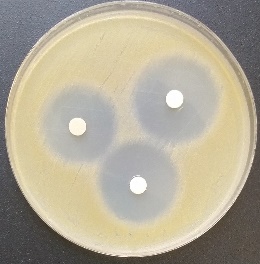 | 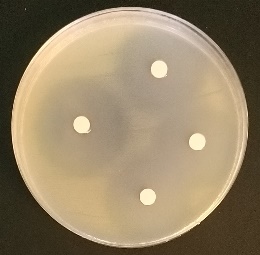 |  |  |
| 8 | C/SiNPs-2.0%ClPh | 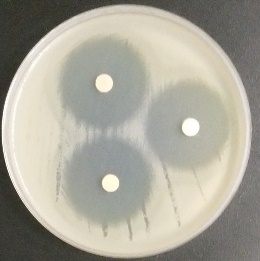 | 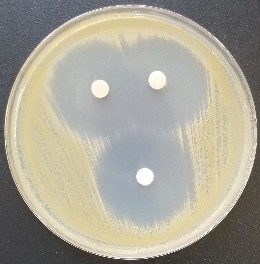 | 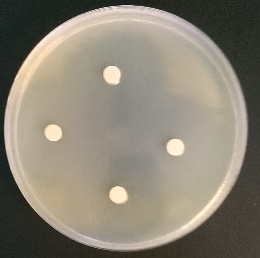 |  |  |
| 9 | C/0.125%ClPh | 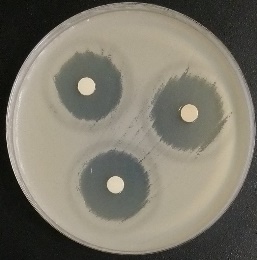 | 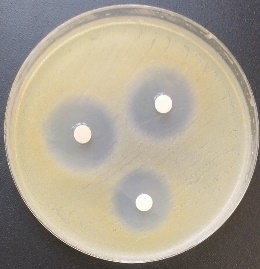 | 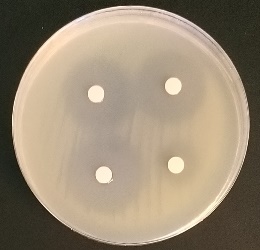 |  |  |
| 10 | C/0.25%ClPh | 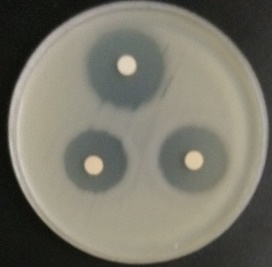 | 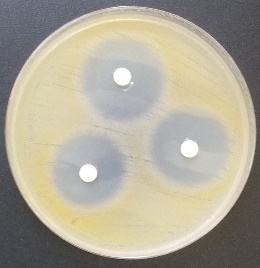 | 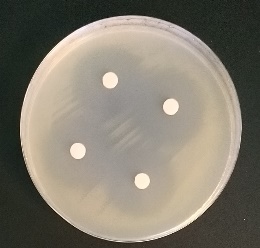 |  |  |
| 11 | C/0.5%ClPh | 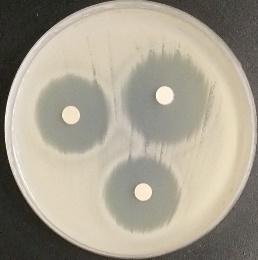 | 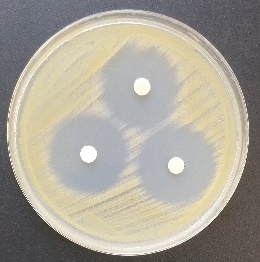 | 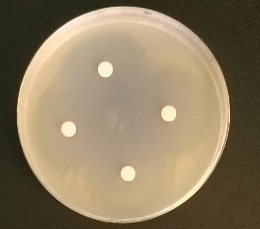 |  |  |
| 12 | C/2.0%ClPh | 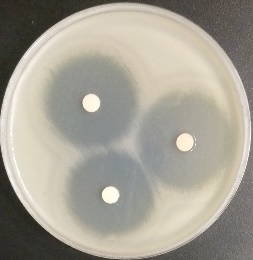 | 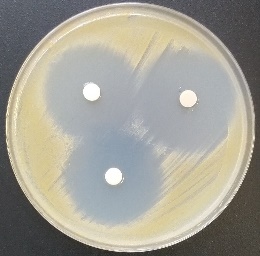 | 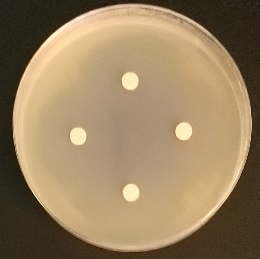 |  |  |
| 13 | Detreomycin 2.0% | 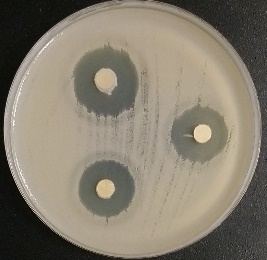 | 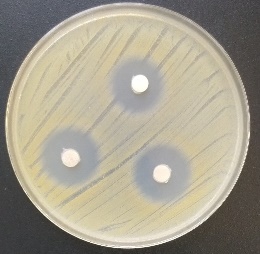 | 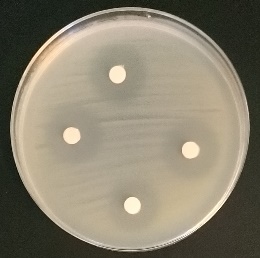 | 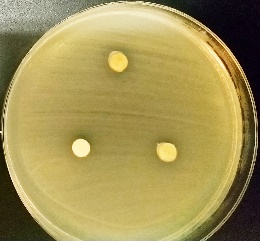 | 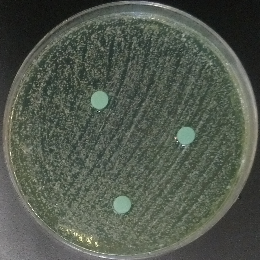 |

C/ - carbopol based formulation; AuNPs – gold nanoparticles; SiNPs – silica nanoparticles, ClPh - chloramphenicol


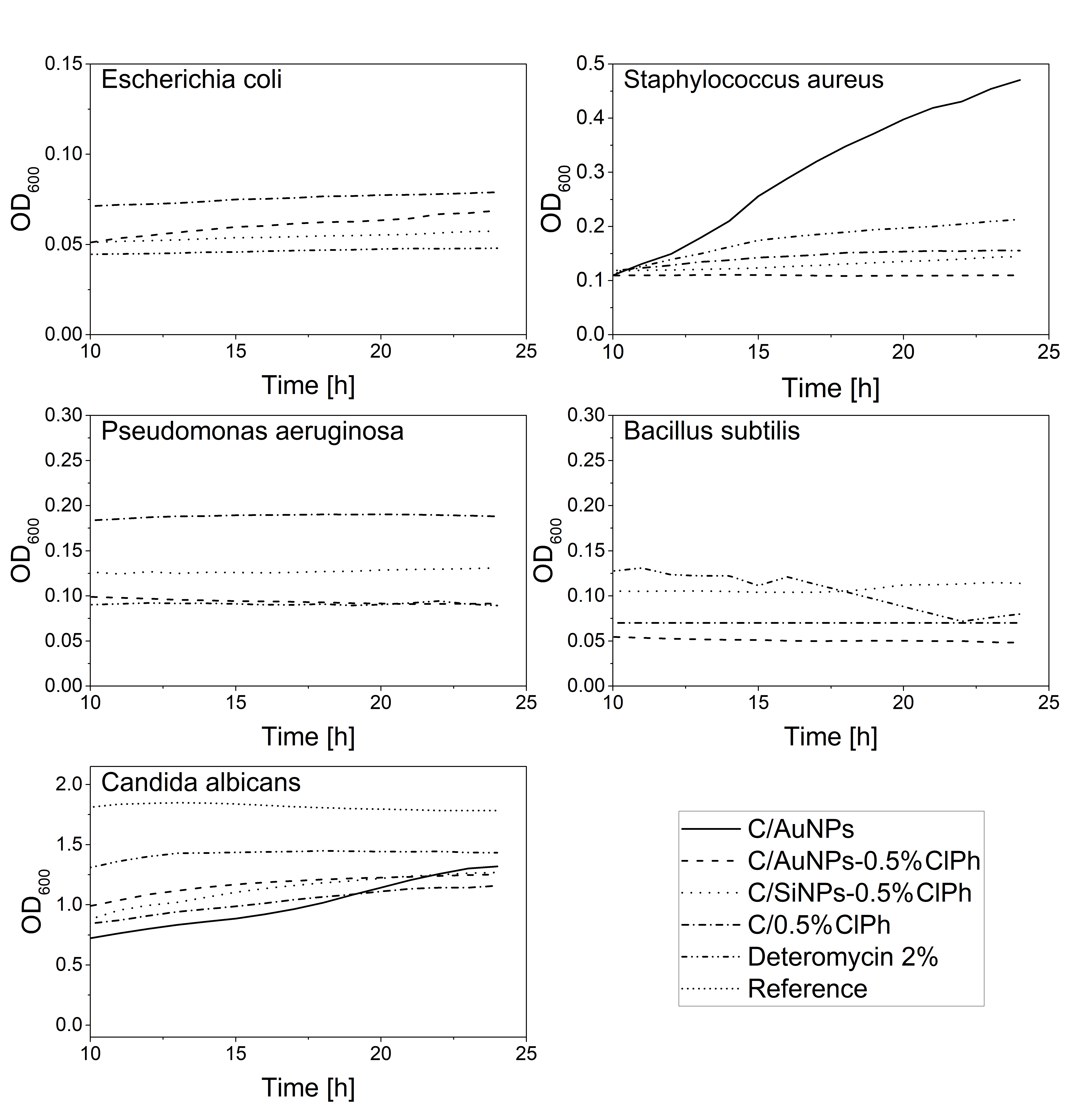


**Figure S3.** Microorganisms growth kinetics tested for selected formulations (the faintly visible area from figure 6)

C/0.5%ClPh – carbopol formulation based only on chloramphenicol (without the silica or nanogold carriers); C/SiNPs-0.5%ClPh - carbopol formulation based on SiNPs carrier and chloramphenicol; C/AuNPs-0.5%ClPh - carbopol formulation based on AuNPs carrier and chloramphenicol;

**Table S13.** Results of Fisher’s LSD Test for *B. subtilis* OD600 of tested formulations

| No.g | LSD Test; variable B.subtilis OD600  Probabilities for Post Hoc Tests  Error: Between MS = .01517. df = 336.00 | | | | | | | | | | | | | | |
| --- | --- | --- | --- | --- | --- | --- | --- | --- | --- | --- | --- | --- | --- | --- | --- |
|  | Formulation | C/  AuNPs | C/AuNPs-  0.125%ClPh | C/AuNPs-  0.25%ClPh | C/AuNPs-  0.5%ClPh | C/SiNPs-  0.125%  ClPh | C/SiNPs-  0.25%ClPh | C/SiNPs-  0.5%ClPh | C/SiNPs-  2.0%ClPh | C/0.125%  ClPh | C/0.25%  ClPh | C/0.5%  ClPh | C/2.0%  ClPh | Detreomycin 2.0% | control |
| 1 | C/AuNPs |  | 0.0000 | 0.0000 | 0.0000 | 0.0000 | 0.0000 | 0.0000 | 0.0000 | 0.0000 | 0.0000 | 0.0000 | 0.0000 | 0.0000 | 0.0000 |
| 2 | C/AuNPs-0.125%ClPh | 0.0000 |  | 0.7322 | 0.0732 | 0.8462 | 0.9169 | 0.6872 | 0.2775 | 0.6734 | 0.7924 | 0.2058 | 0.1797 | 0.7453 | 0.0000 |
| 3 | C/AuNPs-0.25%ClPh | 0.0000 | 0.7322 |  | 0.1467 | 0.5918 | 0.8119 | 0.9518 | 0.1535 | 0.9369 | 0.9370 | 0.3555 | 0.0925 | 0.9861 | 0.0000 |
| 4 | C/AuNPs-0.5%ClPh | 0.0000 | 0.0732 | 0.1467 |  | 0.0473 | 0.0914 | 0.1642 | 0.0042 | 0.1699 | 0.1260 | 0.5969 | 0.0018 | 0.1419 | 0.0000 |
| 5 | C/SiNPs-0.125%ClPh | 0.0000 | 0.8462 | 0.5918 | 0.0473 |  | 0.7655 | 0.5508 | 0.3721 | 0.5383 | 0.6476 | 0.1447 | 0.2508 | 0.6040 | 0.0000 |
| 6 | C/SiNPs-0.25%ClPh | 0.0000 | 0.9169 | 0.8119 | 0.0914 | 0.7655 |  | 0.7654 | 0.2340 | 0.7511 | 0.8737 | 0.2455 | 0.1483 | 0.8255 | 0.0000 |
| 7 | C/SiNPs-0.5%ClPh | 0.0000 | 0.6872 | 0.9518 | 0.1642 | 0.5508 | 0.7654 |  | 0.1370 | 0.9850 | 0.8891 | 0.3878 | 0.0815 | 0.9379 | 0.0000 |
| 8 | C/SiNPs-2.0%ClPh | 0.0000 | 0.2775 | 0.1535 | 0.0042 | 0.3721 | 0.2340 | 0.1370 |  | 0.1321 | 0.1775 | 0.0191 | 0.7975 | 0.1586 | 0.0000 |
| 9 | C/0.125%ClPh | 0.0000 | 0.6734 | 0.9369 | 0.1699 | 0.5383 | 0.7511 | 0.9850 | 0.1321 |  | 0.8742 | 0.3982 | 0.0782 | 0.9230 | 0.0000 |
| 10 | C/0.25%ClPh | 0.0000 | 0.7924 | 0.9370 | 0.1260 | 0.6476 | 0.8737 | 0.8891 | 0.1775 | 0.8742 |  | 0.3159 | 0.1088 | 0.9509 | 0.0000 |
| 11 | C/0.5%ClPh | 0.0000 | 0.2058 | 0.3555 | 0.5969 | 0.1447 | 0.2455 | 0.3878 | 0.0191 | 0.3982 | 0.3159 |  | 0.0094 | 0.3465 | 0.0000 |
| 12 | C/2.0%ClPh | 0.0000 | 0.1797 | 0.0925 | 0.0018 | 0.2508 | 0.1483 | 0.0815 | 0.7975 | 0.0782 | 0.1088 | 0.0094 |  | 0.0959 | 0.0000 |
| 13 | Detreomycin 2.0% | 0.0000 | 0.7453 | 0.9861 | 0.1419 | 0.6040 | 0.8255 | 0.9379 | 0.1586 | 0.9230 | 0.9509 | 0.3465 | 0.0959 |  | 0.0000 |
| 14 | control | 0.0000 | 0.0000 | 0.0000 | 0.0000 | 0.0000 | 0.0000 | 0.0000 | 0.0000 | 0.0000 | 0.0000 | 0.0000 | 0.0000 | 0.0000 |  |

C/ - carbopol based formulation; AuNPs – gold nanoparticles; SiNPs – silica nanoparticles, ClPh - chloramphenicol

**Table S14.** Results of Fisher’s LSD Test for *S. aureus* OD600 of tested formulations

| No..g | LSD Test; variable S.aureus OD600  Probabilities for Post Hoc Tests  Error: Between MS = .01573. df = 336.00 | | | | | | | | | | | | | | |
| --- | --- | --- | --- | --- | --- | --- | --- | --- | --- | --- | --- | --- | --- | --- | --- |
|  | Formulation | C/  AuNPs | C/AuNPs-  0.125%ClPh | C/AuNPs-  0.25%ClPh | C/AuNPs-  0.5%ClPh | C/SiNPs-  0.125%  ClPh | C/SiNPs-  0.25%ClPh | C/SiNPs-  0.5%ClPh | C/SiNPs-  2.0%ClPh | C/0.125%  ClPh | C/0.25%  ClPh | C/0.5%  ClPh | C/2.0%  ClPh | Detreomycin 2.0% | control |
| 1 | C/AuNPs |  | 0.0000 | 0.0000 | 0.0000 | 0.0000 | 0.0000 | 0.0000 | 0.0000 | 0.0000 | 0.0000 | 0.0000 | 0.0000 | 0.0000 | 0.0000 |
| 2 | C/AuNPs-0.125%ClPh | 0.0000 |  | 0.6889 | 0.1009 | 0.2226 | 0.8497 | 0.4231 | 0.0370 | 0.6660 | 0.1106 | 0.5868 | 0.0048 | 0.0666 | 0.0000 |
| 3 | C/AuNPs-0.25%ClPh | 0.0000 | 0.6889 |  | 0.2143 | 0.1056 | 0.8329 | 0.6885 | 0.0912 | 0.9750 | 0.2314 | 0.3454 | 0.0154 | 0.1509 | 0.0000 |
| 4 | C/AuNPs-0.5%ClPh | 0.0000 | 0.1009 | 0.2143 |  | 0.0044 | 0.1465 | 0.3998 | 0.6533 | 0.2260 | 0.9640 | 0.0293 | 0.2345 | 0.8453 | 0.0000 |
| 5 | C/SiNPs-0.125%ClPh | 0.0000 | 0.2226 | 0.1056 | 0.0044 |  | 0.1590 | 0.0438 | 0.0010 | 0.0991 | 0.0051 | 0.4984 | 0.0001 | 0.0024 | 0.0000 |
| 6 | C/SiNPs-0.25%ClPh | 0.0000 | 0.8497 | 0.8329 | 0.1465 | 0.1590 |  | 0.5407 | 0.0576 | 0.8086 | 0.1594 | 0.4637 | 0.0085 | 0.0997 | 0.0000 |
| 7 | C/SiNPs-0.5%ClPh | 0.0000 | 0.4231 | 0.6885 | 0.3998 | 0.0438 | 0.5407 |  | 0.1970 | 0.7117 | 0.4256 | 0.1792 | 0.0427 | 0.2999 | 0.0000 |
| 8 | C/SiNPs-2.0%ClPh | 0.0000 | 0.0370 | 0.0912 | 0.6533 | 0.0010 | 0.0576 | 0.1970 |  | 0.0973 | 0.6211 | 0.0087 | 0.4590 | 0.7994 | 0.0000 |
| 9 | C/0.125%ClPh | 0.0000 | 0.6660 | 0.9750 | 0.2260 | 0.0991 | 0.8086 | 0.7117 | 0.0973 |  | 0.2437 | 0.3297 | 0.0168 | 0.1600 | 0.0000 |
| 10 | C/0.25%ClPh | 0.0000 | 0.1106 | 0.2314 | 0.9640 | 0.0051 | 0.1594 | 0.4256 | 0.6211 | 0.2437 |  | 0.0328 | 0.2172 | 0.8101 | 0.0000 |
| 11 | C/0.5%ClPh | 0.0000 | 0.5868 | 0.3454 | 0.0293 | 0.4984 | 0.4637 | 0.1792 | 0.0087 | 0.3297 | 0.0328 |  | 0.0008 | 0.0177 | 0.0000 |
| 12 | C/2.0%ClPh | 0.0000 | 0.0048 | 0.0154 | 0.2345 | 0.0001 | 0.0085 | 0.0427 | 0.4590 | 0.0168 | 0.2172 | 0.0008 |  | 0.3201 | 0.0000 |
| 13 | Detreomycin 2.0% | 0.0000 | 0.0666 | 0.1509 | 0.8453 | 0.0024 | 0.0997 | 0.2999 | 0.7994 | 0.1600 | 0.8101 | 0.0177 | 0.3201 |  | 0.0000 |
| 14 | control | 0.0000 | 0.0000 | 0.0000 | 0.0000 | 0.0000 | 0.0000 | 0.0000 | 0.0000 | 0.0000 | 0.0000 | 0.0000 | 0.0000 | 0.0000 |  |

C/ - carbopol based formulation; AuNPs – gold nanoparticles; SiNPs – silica nanoparticles, ClPh - chloramphenicol

**Table S15.** Results of Fisher’s LSD Test for *E. coli* OD600 of tested formulations

| No. | LSD Test; variable E.coli OD600  Probabilities for Post Hoc Tests  Error: Between MS = .01058. df = 336.00 | | | | | | | | | | | | | | |
| --- | --- | --- | --- | --- | --- | --- | --- | --- | --- | --- | --- | --- | --- | --- | --- |
|  | Formulation | C/  AuNPs | C/AuNPs-  0.125%  ClPh | C/AuNPs-  0.25%ClPh | C/AuNPs-  0.5%ClPh | C/SiNPs-  0.125%  ClPh | C/SiNPs-  0.25%ClPh | C/SiNPs-  0.5%ClPh | C/SiNPs-  2.0%ClPh | C/0.125%  ClPh | C/0.25%  ClPh | C/0.5%  ClPh | C/2.0%  ClPh | Detreomycin 2.0% | control |
| 1 | C/AuNPs |  | 0.0000 | 0.0000 | 0.0000 | 0.0000 | 0.0000 | 0.0000 | 0.0000 | 0.0000 | 0.0000 | 0.0000 | 0.0000 | 0.0000 | 0.0000 |
| 2 | C/AuNPs-0.125%ClPh | 0.0000 |  | 0.7672 | 0.5378 | 0.7310 | 0.7431 | 0.4944 | 0.8882 | 0.6883 | 0.7466 | 0.9875 | 0.8852 | 0.3598 | 0.0000 |
| 3 | C/AuNPs-0.25%ClPh | 0.0000 | 0.7672 |  | 0.7488 | 0.9619 | 0.9747 | 0.6984 | 0.8764 | 0.9163 | 0.9784 | 0.7552 | 0.8794 | 0.5352 | 0.0000 |
| 4 | C/AuNPs-0.5%ClPh | 0.0000 | 0.5378 | 0.7488 |  | 0.7853 | 0.7730 | 0.9463 | 0.6343 | 0.8297 | 0.7694 | 0.5275 | 0.6370 | 0.7642 | 0.0000 |
| 5 | C/SiNPs-0.125%ClPh | 0.0000 | 0.7310 | 0.9619 | 0.7853 |  | 0.9872 | 0.7341 | 0.8389 | 0.9543 | 0.9835 | 0.7192 | 0.8419 | 0.5671 | 0.0000 |
| 6 | C/SiNPs-0.25%ClPh | 0.0000 | 0.7431 | 0.9747 | 0.7730 | 0.9872 |  | 0.7220 | 0.8515 | 0.9415 | 0.9963 | 0.7313 | 0.8545 | 0.5563 | 0.0000 |
| 7 | C/SiNPs-0.5%ClPh | 0.0000 | 0.4944 | 0.6984 | 0.9463 | 0.7341 | 0.7220 |  | 0.5872 | 0.7776 | 0.7185 | 0.4845 | 0.5898 | 0.8160 | 0.0000 |
| 8 | C/SiNPs-2.0%ClPh | 0.0000 | 0.8882 | 0.8764 | 0.6343 | 0.8389 | 0.8515 | 0.5872 |  | 0.7944 | 0.8551 | 0.8759 | 0.9970 | 0.4381 | 0.0000 |
| 9 | C/0.125%ClPh | 0.0000 | 0.6883 | 0.9163 | 0.8297 | 0.9543 | 0.9415 | 0.7776 | 0.7944 |  | 0.9378 | 0.6768 | 0.7973 | 0.6065 | 0.0000 |
| 10 | C/0.25%ClPh | 0.0000 | 0.7466 | 0.9784 | 0.7694 | 0.9835 | 0.9963 | 0.7185 | 0.8551 | 0.9378 |  | 0.7348 | 0.8581 | 0.5532 | 0.0000 |
| 11 | C/0.5%ClPh | 0.0000 | 0.9875 | 0.7552 | 0.5275 | 0.7192 | 0.7313 | 0.4845 | 0.8759 | 0.6768 | 0.7348 |  | 0.8729 | 0.3517 | 0.0000 |
| 12 | C/2.0%ClPh | 0.0000 | 0.8852 | 0.8794 | 0.6370 | 0.8419 | 0.8545 | 0.5898 | 0.9970 | 0.7973 | 0.8581 | 0.8729 |  | 0.4403 | 0.0000 |
| 13 | Detreomycin 2.0% | 0.0000 | 0.3598 | 0.5352 | 0.7642 | 0.5671 | 0.5563 | 0.8160 | 0.4381 | 0.6065 | 0.5532 | 0.3517 | 0.4403 |  | 0.0000 |
| 14 | control | 0.0000 | 0.0000 | 0.0000 | 0.0000 | 0.0000 | 0.0000 | 0.0000 | 0.0000 | 0.0000 | 0.0000 | 0.0000 | 0.0000 | 0.0000 |  |

C/ - carbopol based formulation; AuNPs – gold nanoparticles; SiNPs – silica nanoparticles, ClPh - chloramphenicol

**Table S16.** Results of Fisher’s LSD Test for *P. aeruginosa* OD600 of tested formulations

| No.g | LSD Test; variable P.aeruginosa OD600  Probabilities for Post Hoc Tests  Error: Between MS = .03443. df = 336.00 | | | | | | | | | | | | | | |
| --- | --- | --- | --- | --- | --- | --- | --- | --- | --- | --- | --- | --- | --- | --- | --- |
|  | Formulation | C/  AuNPs | C/AuNPs-  0.125%ClPh | C/AuNPs-  0.25%ClPh | C/AuNPs-  0.5%ClPh | C/SiNPs-  0.125%  ClPh | C/SiNPs-  0.25%ClPh | C/SiNPs-  0.5%ClPh | C/SiNPs-  2.0%ClPh | C/0.125%  ClPh | C/0.25%  ClPh | C/0.5%  ClPh | C/2.0%  ClPh | Detreomycin 2.0% | control |
| 1 | C/AuNPs |  | 0.2797 | 0.0460 | 0.0382 | 0.0607 | 0.0437 | 0.0718 | 0.0695 | 0.0308 | 0.0146 | 0.0655 | 0.2770 | 0.1222 | 0.0000 |
| 2 | C/AuNPs-0.125%ClPh | 0.2797 |  | 0.3584 | 0.3190 | 0.4246 | 0.3471 | 0.4697 | 0.4609 | 0.2785 | 0.1705 | 0.4447 | 0.9950 | 0.6409 | 0.0000 |
| 3 | C/AuNPs-0.25%ClPh | 0.0460 | 0.3584 |  | 0.9377 | 0.9044 | 0.9825 | 0.8447 | 0.8560 | 0.8684 | 0.6503 | 0.8773 | 0.3616 | 0.6509 | 0.0000 |
| 4 | C/AuNPs-0.5%ClPh | 0.0382 | 0.3190 | 0.9377 |  | 0.8428 | 0.9551 | 0.7841 | 0.7952 | 0.9302 | 0.7075 | 0.8161 | 0.3221 | 0.5957 | 0.0000 |
| 5 | C/SiNPs-0.125%ClPh | 0.0607 | 0.4246 | 0.9044 | 0.8428 |  | 0.8871 | 0.9396 | 0.9511 | 0.7750 | 0.5664 | 0.9727 | 0.4282 | 0.7396 | 0.0000 |
| 6 | C/SiNPs-0.25%ClPh | 0.0437 | 0.3471 | 0.9825 | 0.9551 | 0.8871 |  | 0.8276 | 0.8389 | 0.8857 | 0.6661 | 0.8601 | 0.3503 | 0.6352 | 0.0000 |
| 7 | C/SiNPs-0.5%ClPh | 0.0718 | 0.4697 | 0.8447 | 0.7841 | 0.9396 | 0.8276 |  | 0.9885 | 0.7177 | 0.5163 | 0.9669 | 0.4736 | 0.7974 | 0.0000 |
| 8 | C/SiNPs-2.0%ClPh | 0.0695 | 0.4609 | 0.8560 | 0.7952 | 0.9511 | 0.8389 | 0.9885 |  | 0.7285 | 0.5257 | 0.9784 | 0.4647 | 0.7863 | 0.0000 |
| 9 | C/0.125%ClPh | 0.0308 | 0.2785 | 0.8684 | 0.9302 | 0.7750 | 0.8857 | 0.7177 | 0.7285 |  | 0.7736 | 0.7489 | 0.2812 | 0.5365 | 0.0000 |
| 10 | C/0.25%ClPh | 0.0146 | 0.1705 | 0.6503 | 0.7075 | 0.5664 | 0.6661 | 0.5163 | 0.5257 | 0.7736 |  | 0.5435 | 0.1725 | 0.3653 | 0.0000 |
| 11 | C/0.5%ClPh | 0.0655 | 0.4447 | 0.8773 | 0.8161 | 0.9727 | 0.8601 | 0.9669 | 0.9784 | 0.7489 | 0.5435 |  | 0.4484 | 0.7656 | 0.0000 |
| 12 | C/2.0%ClPh | 0.2770 | 0.9950 | 0.3616 | 0.3221 | 0.4282 | 0.3503 | 0.4736 | 0.4647 | 0.2812 | 0.1725 | 0.4484 |  | 0.6454 | 0.0000 |
| 13 | Detreomycin 2.0% | 0.1222 | 0.6409 | 0.6509 | 0.5957 | 0.7396 | 0.6352 | 0.7974 | 0.7863 | 0.5365 | 0.3653 | 0.7656 | 0.6454 |  | 0.0000 |
| 14 | control | 0.0000 | 0.0000 | 0.0000 | 0.0000 | 0.0000 | 0.0000 | 0.0000 | 0.0000 | 0.0000 | 0.0000 | 0.0000 | 0.0000 | 0.0000 |  |

C/ - carbopol based formulation; AuNPs – gold nanoparticles; SiNPs – silica nanoparticles, ClPh - chloramphenicol

**Table S17.** Results of Fisher’s LSD Test for *C.albicans* OD600 of tested formulations

| No. | LSD Test; variable C.albicans OD600  Probabilities for Post Hoc Tests  Error: Between MS = .13908. df = 336.00 | | | | | | | | | | | | | | |
| --- | --- | --- | --- | --- | --- | --- | --- | --- | --- | --- | --- | --- | --- | --- | --- |
|  | Formulation | C/  AuNPs | C/AuNPs-  0.125%ClPh | C/AuNPs-  0.25%ClPh | C/AuNPs-  0.5%ClPh | C/SiNPs-  0.125%  ClPh | C/SiNPs-  0.25%ClPh | C/SiNPs-  0.5%ClPh | C/SiNPs-  2.0%  ClPh | C/0.125%  ClPh | C/0.25%  ClPh | C/0.5%  ClPh | C/2.0%  ClPh | Detreomycin 2.0% | control |
| 1 | C/AuNPs |  | 0.4929 | 0.7215 | 0.2869 | 0.1183 | 0.0213 | 0.4901 | 0.9371 | 0.8259 | 0.9059 | 0.9215 | 0.0659 | 0.0023 | 0.0000 |
| 2 | C/AuNPs-0.125%ClPh | 0.4929 |  | 0.7418 | 0.7040 | 0.3797 | 0.1047 | 0.9964 | 0.4446 | 0.6413 | 0.5703 | 0.5571 | 0.2475 | 0.0176 | 0.0000 |
| 3 | C/AuNPs-0.25%ClPh | 0.7215 | 0.7418 |  | 0.4782 | 0.2274 | 0.0512 | 0.7384 | 0.6633 | 0.8915 | 0.8117 | 0.7965 | 0.1377 | 0.0069 | 0.0000 |
| 4 | C/AuNPs-0.5%ClPh | 0.2869 | 0.7040 | 0.4782 |  | 0.6178 | 0.2135 | 0.7073 | 0.2528 | 0.3979 | 0.3436 | 0.3337 | 0.4370 | 0.0456 | 0.0000 |
| 5 | C/SiNPs-0.125%ClPh | 0.1183 | 0.3797 | 0.2274 | 0.6178 |  | 0.4556 | 0.3821 | 0.1009 | 0.1792 | 0.1486 | 0.1432 | 0.7806 | 0.1328 | 0.0000 |
| 6 | C/SiNPs-0.25%ClPh | 0.0213 | 0.1047 | 0.0512 | 0.2135 | 0.4556 |  | 0.1057 | 0.0173 | 0.0371 | 0.0289 | 0.0275 | 0.6399 | 0.4480 | 0.0001 |
| 7 | C/SiNPs-0.5%ClPh | 0.4901 | 0.9964 | 0.7384 | 0.7073 | 0.3821 | 0.1057 |  | 0.4419 | 0.6381 | 0.5673 | 0.5541 | 0.2493 | 0.0178 | 0.0000 |
| 8 | C/SiNPs-2.0%ClPh | 0.9371 | 0.4446 | 0.6633 | 0.2528 | 0.1009 | 0.0173 | 0.4419 |  | 0.7650 | 0.8437 | 0.8591 | 0.0552 | 0.0018 | 0.0000 |
| 9 | C/0.125%ClPh | 0.8259 | 0.6413 | 0.8915 | 0.3979 | 0.1792 | 0.0371 | 0.6381 | 0.7650 |  | 0.9189 | 0.9034 | 0.1052 | 0.0046 | 0.0000 |
| 10 | C/0.25%ClPh | 0.9059 | 0.5703 | 0.8117 | 0.3436 | 0.1486 | 0.0289 | 0.5673 | 0.8437 | 0.9189 |  | 0.9843 | 0.0852 | 0.0034 | 0.0000 |
| 11 | C/0.5%ClPh | 0.9215 | 0.5571 | 0.7965 | 0.3337 | 0.1432 | 0.0275 | 0.5541 | 0.8591 | 0.9034 | 0.9843 |  | 0.0817 | 0.0032 | 0.0000 |
| 12 | C/2.0%ClPh | 0.0659 | 0.2475 | 0.1377 | 0.4370 | 0.7806 | 0.6399 | 0.2493 | 0.0552 | 0.1052 | 0.0852 | 0.0817 |  | 0.2203 | 0.0000 |
| 13 | Detreomycin 2.0% | 0.0023 | 0.0176 | 0.0069 | 0.0456 | 0.1328 | 0.4480 | 0.0178 | 0.0018 | 0.0046 | 0.0034 | 0.0032 | 0.2203 |  | 0.0011 |
| 14 | control | 0.0000 | 0.0000 | 0.0000 | 0.0000 | 0.0000 | 0.0001 | 0.0000 | 0.0000 | 0.0000 | 0.0000 | 0.0000 | 0.0000 | 0.0011 |  |

C/ - carbopol based formulation; AuNPs – gold nanoparticles; SiNPs – silica nanoparticles, ClPh - chloramphenicol
